# Drug-target identification in COVID-19 disease mechanisms using computational systems biology approaches

**DOI:** 10.3389/fimmu.2023.1282859

**Published:** 2024-02-13

**Authors:** Anna Niarakis, Marek Ostaszewski, Alexander Mazein, Inna Kuperstein, Martina Kutmon, Marc E. Gillespie, Akira Funahashi, Marcio Luis Acencio, Ahmed Hemedan, Michael Aichem, Karsten Klein, Tobias Czauderna, Felicia Burtscher, Takahiro G. Yamada, Yusuke Hiki, Noriko F. Hiroi, Finterly Hu, Nhung Pham, Friederike Ehrhart, Egon L. Willighagen, Alberto Valdeolivas, Aurelien Dugourd, Francesco Messina, Marina Esteban-Medina, Maria Peña-Chilet, Kinza Rian, Sylvain Soliman, Sara Sadat Aghamiri, Bhanwar Lal Puniya, Aurélien Naldi, Tomáš Helikar, Vidisha Singh, Marco Fariñas Fernández, Viviam Bermudez, Eirini Tsirvouli, Arnau Montagud, Vincent Noël, Miguel Ponce-de-Leon, Dieter Maier, Angela Bauch, Benjamin M. Gyori, John A. Bachman, Augustin Luna, Janet Piñero, Laura I. Furlong, Irina Balaur, Adrien Rougny, Yohan Jarosz, Rupert W. Overall, Robert Phair, Livia Perfetto, Lisa Matthews, Devasahayam Arokia Balaya Rex, Marija Orlic-Milacic, Luis Cristobal Monraz Gomez, Bertrand De Meulder, Jean Marie Ravel, Bijay Jassal, Venkata Satagopam, Guanming Wu, Martin Golebiewski, Piotr Gawron, Laurence Calzone, Jacques S. Beckmann, Chris T. Evelo, Peter D’Eustachio, Falk Schreiber, Julio Saez-Rodriguez, Joaquin Dopazo, Martin Kuiper, Alfonso Valencia, Olaf Wolkenhauer, Hiroaki Kitano, Emmanuel Barillot, Charles Auffray, Rudi Balling, Reinhard Schneider, the COVID-19 Disease Map Community

**Affiliations:** ^1^ Université Paris-Saclay, Laboratoire Européen de Recherche pour la Polyarthrite rhumatoïde - Genhotel, Univ Evry, Evry, France; ^2^ Lifeware Group, Inria, Saclay-île de France, Palaiseau, France; ^3^ Luxembourg Centre for Systems Biomedicine, University of Luxembourg, Esch-sur-Alzette, Luxembourg; ^4^ Institut Curie, P.S.L. Research University, Paris, France; ^5^ INSERM, Paris, France; ^6^ MINES ParisTech, PSL Research University, CBIO-Centre for Computational Biology, Paris, France; ^7^ Maastricht Centre for Systems Biology (MaCSBio), Maastricht University, Maastricht, Netherlands; ^8^ Ontario Institute for Cancer Research, Toronto, ON, Canada; ^9^ St. John’s University, Queens, NY, United States; ^10^ Department of Biosciences and Informatics, Keio University, Kanagawa, Japan; ^11^ Department of Computer and Information Science, University of Konstanz, Konstanz, Germany; ^12^ Faculty of Applied Computer Sciences & Biosciences, University of Applied Sciences Mittweida, Mittweida, Germany; ^13^ Center for Biosciences and Informatics, Graduate School of Fundamental Science and Technology, Keio University, Kanagawa, Japan; ^14^ Faculty of Creative Engineering, Kanagawa Institute of Technology, Kanagawa, Japan; ^15^ Keio University School of Medicine, Tokyo, Japan; ^16^ Department of Bioinformatics - BiGCaT, NUTRIM, Maastricht University, Maastricht, Netherlands; ^17^ Institute for Computational Biomedicine, Heidelberg University, Faculty of Medicine, Heidelberg University Hospital, Bioquant, Heidelberg, Germany; ^18^ Department of Epidemiology, Preclinical Research and Advanced Diagnostic, National Institute for Infectious Diseases’ Lazzaro Spallanzani’ - IRCCS, Rome, Italy; ^19^ Computational Medicine Platform, Andalusian Public Foundation Progress and Health-FPS, Sevilla, Spain; ^20^ Computational Systems Medicine, Institute of Biomedicine of Seville (IBIS), Hospital Virgen del Rocío, Sevilla, Spain; ^21^ Bioinformatics in Rare Diseases (BiER), Centro de Investigación Biomédica en Red de Enfermedades Raras (CIBERER), FPS, Hospital Virgen del Rocio, Seville, Spain; ^22^ Department of Biochemistry, University of Nebraska-Lincoln, Lincoln, NE, United States; ^23^ Department of Biology, Norwegian University of Science and Technology, Trondheim, Norway; ^24^ Barcelona Supercomputing Center (BSC.), Barcelona, Spain; ^25^ Labvantage-Biomax GmbH, Planegg, Germany; ^26^ Harvard Medical School, Laboratory of Systems Pharmacology, Boston, MA, United States; ^27^ Computational Biology Branch, National Library of Medicine, Bethesda, MD, United States; ^28^ Department of Systems Biology, Harvard Medical School, Boston, MA, United States; ^29^ Medbioinformatics Solutions SL, Barcelona, Spain; ^30^ Research Programme on Biomedical Informatics (GRIB), Hospital del Mar Medical Research Institute (IMIM), Dept. of Medicine and Life Sciences, Universitat Pompeu Fabra (UPF), Barcelona, Spain; ^31^ Biotechnology Research Institute for Drug Discovery, National Institute of Advanced Industrial Science and Technology (AIST), Aomi, Tokyo, Japan; ^32^ Com. Bio Big Data Open Innovation Lab. (CBBD-OIL), AIST, Aomi, Tokyo, Japan; ^33^ Institute for Biology, Humboldt University of Berlin, Berlin, Germany; ^34^ Integrative Bioinformatics, Inc., Mountain View, CA, United States; ^35^ Department of Biology and Biotechnology Charles Darwin, Sapienza University of Rome, Rome, Italy; ^36^ Department of Biochemistry & Molecular Pharmacology, NYU. Langone Medical Center, New York, NY, United States; ^37^ Centre for Integrative Omics Data Science, Yenepoya (Deemed to be University), Derlakatte, Mangalore, India; ^38^ Association E.I.S.B.M, Brignais, France; ^39^ Frankfurt Institute for Advanced Studies, Johann Wolfgang Goethe-Universität Frankfurt, Frankfurt am Main, Germany; ^40^ Oregon Health Sciences University, Portland, OR, United States; ^41^ Heidelberg Institute for Theoretical Studies (HITS), Heidelberg, Germany; ^42^ University of Lausanne, Lausanne, Switzerland; ^43^ Faculty of Information Technology, Monash University, Clayton, Victoria, VIC, Australia; ^44^ FPS/ELIXIR-es, Hospital Virgen del Rocío, Sevilla, Spain; ^45^ I.C.R.E.A., Pg. Lluís Companys 23, Barcelona, Spain; ^46^ Department of Systems Biology & Bioinformatics, University of Rostock, Rostock, Germany; ^47^ Leibniz Institute for Food Systems Biology, at the Technical University Munich, Munich, Germany; ^48^ Systems Biology Institute, Tokyo, Japan; ^49^ Institute of Molecular Psychiatry, University of Bonn, Bonn, Germany; ^50^ FAIRDOMHub: https://fairdomhub.org/projects/190

**Keywords:** SARS-CoV-2, systems biology, disease maps, mechanistic models, dynamic models, systems medicine, large-scale community effort

## Abstract

**Introduction:**

The COVID-19 Disease Map project is a large-scale community effort uniting 277 scientists from 130 Institutions around the globe. We use high-quality, mechanistic content describing SARS-CoV-2-host interactions and develop interoperable bioinformatic pipelines for novel target identification and drug repurposing.

**Methods:**

Extensive community work allowed an impressive step forward in building interfaces between Systems Biology tools and platforms. Our framework can link biomolecules from omics data analysis and computational modelling to dysregulated pathways in a cell-, tissue- or patient-specific manner. Drug repurposing using text mining and AI-assisted analysis identified potential drugs, chemicals and microRNAs that could target the identified key factors.

**Results:**

Results revealed drugs already tested for anti-COVID-19 efficacy, providing a mechanistic context for their mode of action, and drugs already in clinical trials for treating other diseases, never tested against COVID-19.

**Discussion:**

The key advance is that the proposed framework is versatile and expandable, offering a significant upgrade in the arsenal for virus-host interactions and other complex pathologies.

## 1 Introduction

The COVID-19 global pandemic was caused by the severe acute respiratory syndrome coronavirus 2 (SARS-CoV-2). The novel virus, first identified in December 2019 in China, subsequently spread worldwide. Globally, as of 8 November 2023, there have been over 770 million confirmed cases of COVID-19 and nearly 7 million deaths^1^. As of 5 November 2023, 13 534,474,309 vaccine doses have been administered. On May 5 2023, the World Health Organisation (WHO) declared that COVID-19 was no longer considered a public health emergency, but the pandemic status remains, with the close monitoring of emerging variants^2^. Moreover, the aetiology of the prevalent long COVID syndrome is still unknown. Therefore, the study of potential novel targeted therapies for COVID-19 is still relevant and valuable.

Large-scale community efforts to study molecular mechanisms of SARS-CoV-2 infection, including the COVID-19 Disease Map (C19DMap) project, aim to build an open-access, computable repository of COVID-19 molecular mechanisms (1). The C19DMap comprises forty molecular pathways compliant with systems biology standards, such as SBGN (2) and SBML (3). The pathways were compiled from published COVID-19 research through collective biocuration supported, where possible, by text mining solutions, such as INDRA (4) and AILANI (https://ailani.ai). The C19DMap computational framework is a structure that includes tools and platforms for data integration, analysis, and computational modelling (1, 5) that can be combined with the diagrammatic content.

**Figure 1:**
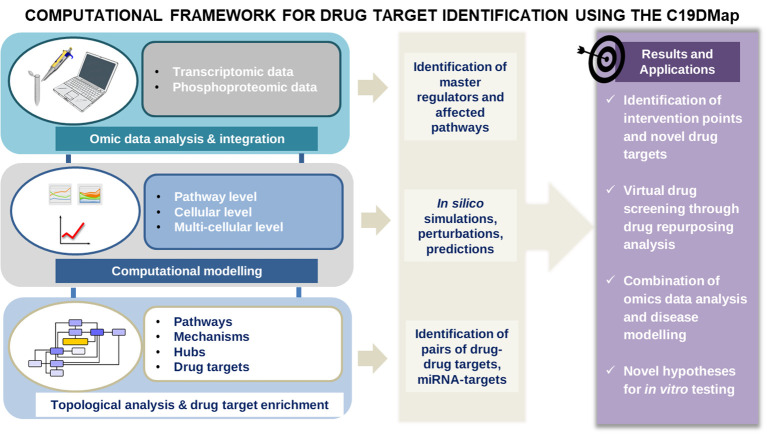
The main workflow of the pipelines was developed to analyse the mechanistic content of the C19Dmap. We used it to suggest intervention points, drug repurposing and novel hypotheses for *in vitro* testing.

The map is an entry point for analytical and modelling workflows to identify actionable targets for novel or repurposed compounds that can mitigate the viral infection or alleviate COVID-19 symptoms. Similar workflows have been used to study immune and chronic diseases (6, 7–9), focusing either on omic data analysis and integration, network analysis, mathematical modelling or drug repurposing (**Figure 1**). This work presents a full range of potential analyses enabled by C19DMap. It outlines how different analytic approaches can be combined meaningfully and impactfully. We use as an example the COVID-19 disease because it is the perfect use case for showcasing start-to-end ways to employ multimodal omic analysis and predictive modelling on a well-curated mechanistic content.

First, we demonstrate how a static diagram can be the template of data integration and computational modelling, leading to predictions and suggestions about the possible outcomes of multiple perturbations in a cell, tissue or even patient-specific manner. Then, we employ text mining and AI-assisted analysis to identify drugs for the retrieved targets, and we suggest selected combinations with predictive efficacy. Finally, we demonstrate the relevance of this work for future pandemic preparedness.

## 2 Results

### 2.1 Multi-omic data analysis

**Figure 2:**
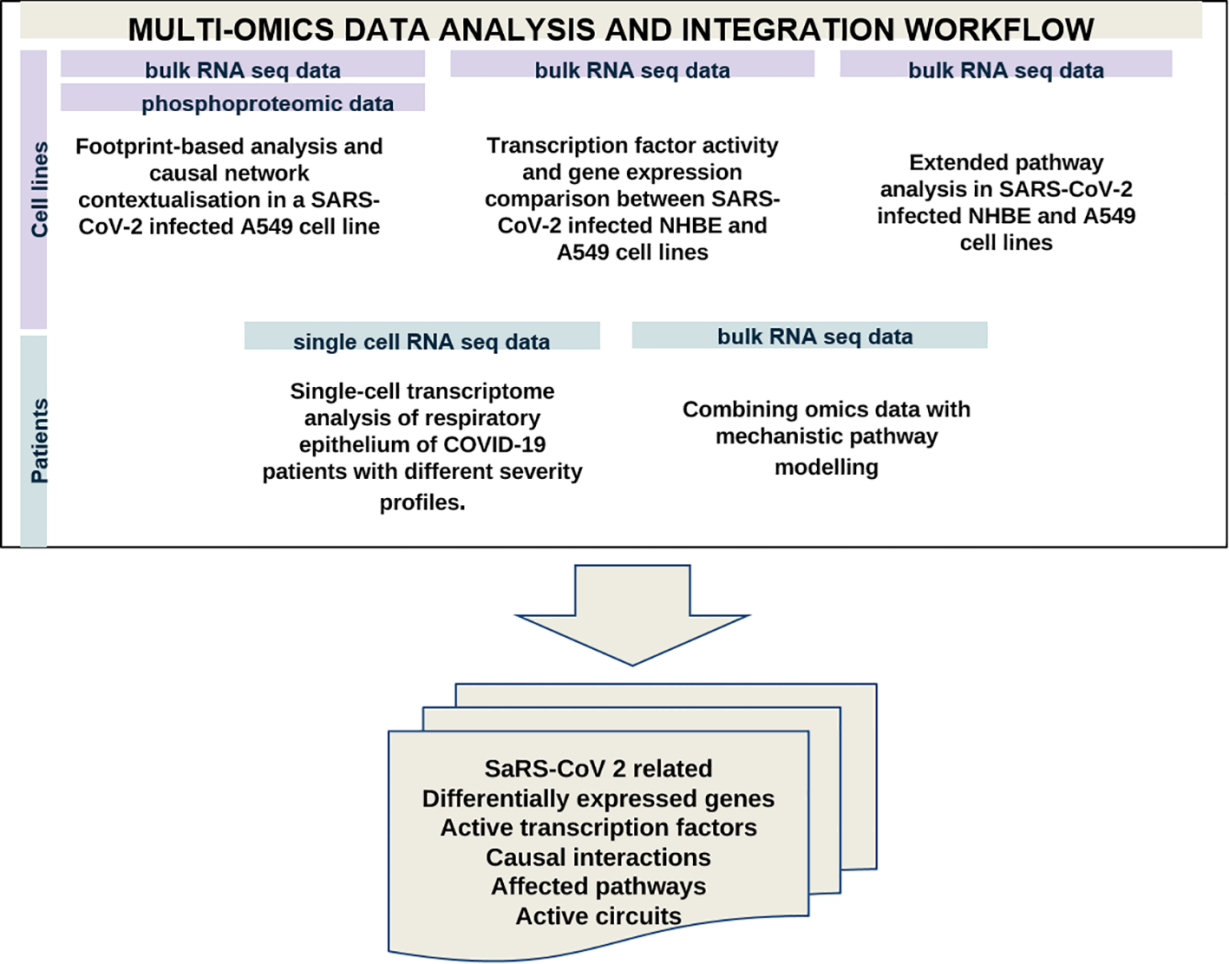
Multi-omics data analysis using available omics data to identify differentially expressed genes, active TFS, causal interactions and affected pathways in samples from cell lines and SARS-CoV-2 patients.

We analysed available omics data (microarray, bulk RNA-seq, scRNA-seq, phosphoproteomic) to identify COVID-19-affected biological processes in cell lines, and patient-derived bronchoalveolar lavage samples and nasopharyngeal swabs. These datasets were not previously used for curating and generating the C19DMap. Identified differentially expressed genes and implicated active transcription factors were then delineated in the C19DMap to determine their functional environment. Finally, mechanistic pathway modelling was applied to assess the impact of the viral infection on relevant cellular functions represented in C19DMap. The methods and tools selected were complementary and brought new insights into SARS-CoV-2 host interactions by combining expression data and the mechanistic content. Footprint-based analysis (6) combines transcriptomic and phosphoproteomic data to identify active transcription factors (TFs). TFs can also be identified by limitless arity multiple testing procedures (10). Both types of analyses could identify TFs already present in the C19DMap and inform on their pathway implication but also reveal new TFs that were not included in the repository. We extended our analyses to the WikiPathways and Reactome repositories, identifying pathways and processes affected by the viral infection. We employed the HiPathia approach (11) that effectively combines RNAseq data with mechanistic diagrams and pathway modelling to expand on patient data and use the available diagrams in predicting active circuits. A small dataset of single-cell RNA data from SARS-CoV-2 patients was also employed to demonstrate the scalability and applicability of the framework (**Figure 2**).

We have performed complementary analyses at the level of cell lines and patients’ samples. **Table 1** recapitulates the type of analysis and approach for the different datasets. We took advantage of the plurality and complementarity of the tools and methodologies developed in the C19DMap community to infer complementary information regarding activated TFs, differentially expressed genes (DEGs), and pathways in the context of SARS-CoV2 infection.

**Table 1:** Type of omics data and analysis performed with the C19DMap repository.

Omics Data	Cell line/patient samples	TF identification	DEG identification	Kinase identification	Pathway identification
*bulk phospho* *proteomic*	*A549*	*Footprint analysis A549 infected/control*	*Footprint analysis A549 infected/control*	*Footprint analysis* *A549 infected/control*	*C19DMap and network inference using CARNIVAL - use of OmniPath resources*
*bulk RNA-seq*	*A549*	*Footprint analysis A549 infected/control*	*Footprint analysis A549 infected/control*	*Footprint analysis* *A549 infected/control*	*C19DMap and network inference using CARNIVAL - use of OmniPath resources &* *Extended pathway analysis (C19DMap+ WikiPathways+Reactome)*
*bulk RNA-seq*	*NHBE*	*LAMP analysis*	*A549 infected/NHBE infected*		*Extended pathway analysis (C19DMap+ WikiPathways+Reactome)*
*scRNA-seq*	*patients (bronchoalveolar samples)*	*sc analysis*	*patients/control*		*C19DMap*
*bulk RNA-seq*	*patients* *(nasal swabs)*	*Hipathia analysis*	*patients/control*		*C19DMap*

#### 2.1.1 Identification of active kinases, TFs, causal interactions, DEGs and affected pathways in SARS-CoV-2 infected cell lines

First, we analyzed bulk RNA seq and phosphoproteomic data from A549 and Normal Human Bronchial Epithelial (NHBE) cell lines (12–14) to identify DEGs, kinases, active TFs and pathways affected in the context of the SARS-CoV-2 infection. We employed complementary tools and approaches to infer the maximum information from the available data. We performed differential expression analysis to identify DEGs and differentially phosphorylated proteins between SARS-CoV-2-infected and mock-treated A549 cells. DEGs were also detected between A549 and NHBE cell lines. Active TFs were identified using two approaches. For the A549 cells for which phosphoproteomic data were available, the Carnival tool (15) with the COSMOS approach (16) was used to contextualise signalling events perturbed during the viral infection and infer a causal network. The best Carnival-inferred causal network connected eight of the top ten deregulated kinases with the top 30 deregulated TFs (TFs; **Supplementary Figure S1A**), including connecting intermediary genes. CARNIVAL takes as input a predefined knowledge network based on OmniPath resources (11) and a series of constraints - top deregulated TFs and kinases in our case, subsequently computing the most likely causal interactions through the resolution of an integer linear programming problem. COSMOS extends this approach to encompass multi-omics data. Regarding the DEGs detected between A549 and NHBE cell lines, limitless arity multiple testing procedures identified the TFs that statistically significantly regulate them (LAMP; **Supplementary Figure S2B**) (10).

The results for A549 cells highlighted four kinases (TBK1, IKBKE, TICAM1, MAPK3), four TFs (IRF3, ATF4, ATF6, SMAD1), and one serine protease (MBTPS1) distributed among seven sub-map diagrams of the C19DMap (**Supplementary Figure S1B**, **Supplementary Table S1**). Activation of the MAP kinases in SARS-CoV-2 infection has been reported previously (17, 18). MAPK3 and SMAD1 take part in TGFbeta family signalling, which may be related to the healing of the damaged lung tissue and the consequent lung fibrosis, which has been reported in COVID-19 (17, 19). Canonical signalling proteins (PIK3CA, BRCA1, and RUNX1) are likely involved in the regulation of cell growth and division (18, 20, 21), while the immune system-related genes TICAM1, TBK1, IKBKE, and IRF3 are found in the pathogen-associated molecular patterns (PAMPs) and Interferon-1 pathways of the C19DMap. Lastly, ATF4, ATF6, and MBTPS1 are part of the Endoplasmic Reticulum (ER) stress pathway. A higher number of TFs and DEGs was detected for A549 than for NHBE cells. Many TFs detected in both cell types were involved in immune response, of which several were present in the C19DMap (IRF3, BACH1, TBP, TCF12, TP53, STAT1, FOS, RELA, NFK1, JUN, STAT2, IRF9, FOSL1), while others, such as ESR1 and KLF6, were novel (**Supplementary Table S2**). The additional highlighted pathways included Interferon lambda signalling, HMOX1 pathway, Pyrimidine deprivation, and kynurenine synthesis.

**Figure 3:**
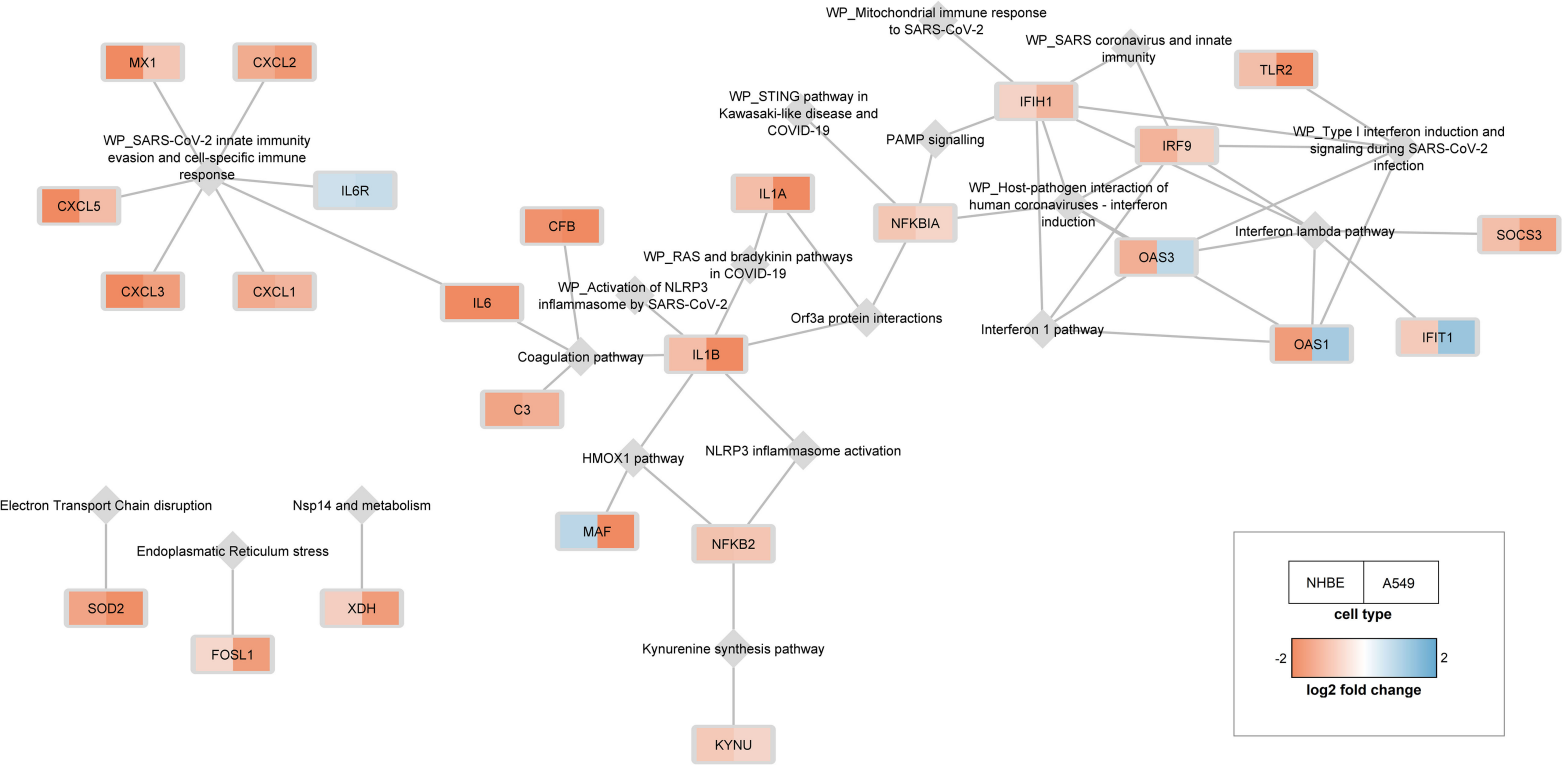
25 genes differentially expressed in both cell lines are linked to 19 pathways. C19DMap pathways are represented as grey diamonds, and shared DEGs are coloured as rectangles following expression fold change.

Using the shared DEGs between the A549 and NHBE cells, and the C19DMap pathways (23 pathways with 657 unique genes), a pathway-gene network was constructed that consisted of 25 genes linked to 19 pathways (**Figure 3**). Central genes in the pathway-gene network were found to be IFIH1 (7 pathways), IL1B (6 pathways), and IRF9 (5 pathways). Interestingly, four genes (OAS1, OAS3, and IFIT1 from the Interferon pathway and MAF from the HMOX1 pathway) had opposite expression profiles in the two cell lines. Many of the shared DEGs (134 out of 159) are not part of the C19DMap, implying that they were not included in the functional studies used to construct the C19DMap, and thus providing an essential resource for future research and curation efforts to understand and map out processes affected by SARS-CoV-2 infection.

#### 2.1.2 Extended pathway analysis in SARS-CoV-2 infected NHBE and A549 cell lines

To enlarge the scope of the analysis and enrich the omics-based pathway analysis, we combined pathways from C19DMap (1), WikiPathways (22), and Reactome (23). The collection included 1,840 human pathways containing 12,037 unique genes in total. We used this extended pathway database to identify altered COVID-19-specific and general molecular pathways in (14) NHBE and A549 cells (**Supplementary Figure S3A**). The analysis revealed 74 and 101 altered pathways in NHBE and A549 cells, respectively, of which 11 were changed in both, including several immune- and metabolism-related pathways (**Supplementary Figure S3B**). Interestingly, SARS-CoV-2-infected NHBE cells showed several altered C19DMap pathways, including interferon and coagulation pathways (**Supplementary Figure S4**), while A549 cells mainly showed changes in general processes, such as cell cycle, DNA mismatch repair, and cholesterol biosynthesis pathways (**Supplementary Figure S5**).

#### 2.1.3 Identification of DEGs, pathways and active circuits in COVID-19 patients’ samples

To investigate if the identified cell-line pathways and TFs were relevant in patients infected with SARS-CoV2, we analyzed scRNA-seq data of bronchoalveolar lavages from nine COVID-19 patients (GSE145926) (24) and epithelial cells isolated from the lungs of nine healthy subjects (GSE160664) (25). Clustering analysis on the entire matrix showed 44 distinct clusters as the best representation of cell types (**Supplementary Figure S6**). Five epithelial cell types were selected by cell sample size and marker genes (26). Differential expression analysis was performed for each cell type between COVID-19 and healthy controls. Among DEGs overexpressed in each cell type in COVID-19 patients (**Supplementary Table S3**), 26 were common to all five lung epithelial cell types (**Supplementary Table S4**). The C19DMap was analysed to evaluate the activation of specific pathways by these 26 overexpressed genes. The most affected pathway was type 1 IFN response (WP4868), with overexpressed IFIH1, OAS1, STAT1, OAS2, OAS3, and IRF7 genes. Several other C19DMap pathways were affected, including NLRP3 inflammasome activation, Interferon lambda pathway, Virus replication cycle, PAMP signalling, TGF-beta signalling, Endoplasmic reticulum stress, Apoptosis pathway, HMOX1 pathway and Renin-Angiotensin pathway.

To combine C19DMap with patient data and expand its utility beyond pathway enrichment, we employed the HiPathia approach (27) that effectively combines RNAseq data with mechanistic pathway modelling. The Hipathia algorithm determines the cell functional profile induced by gene expression changes in the studied condition and supports testing perturbations. HiPathia conceptualises pathways as directed graphs, linking molecular participants through activations and inhibitions, similar to an electrical circuit representation. HiPathia ascribes the activation level to protein nodes in the circuit based on gene expression values of corresponding genes, enhancing understanding of gene expression dynamics in the context of the C19DMap. A public RNAseq dataset of nasopharyngeal swabs from 430 individuals with SARS-CoV-2 and 54 negative controls (26) (GSE152075) and 16 of the 23 C19DMap pathways suitable for the HiPathia algorithm, converted to 145 HiPathia circuits, were used for mechanistic pathway modelling. Of the 145 C19DMap-derived HiPathia circuits, 46 were differentially activated (FDR adjusted p-value < 0.05) (**Supplementary Table S5**). Almost all C19DMap pathways that contained the deregulated circuits showed differential activity between infected and normal cells, confirming the relevance of the C19DMap. Genes central to the activity of each circuit are promising drug target candidates for modulation of downstream processes.

**Figure 4:**
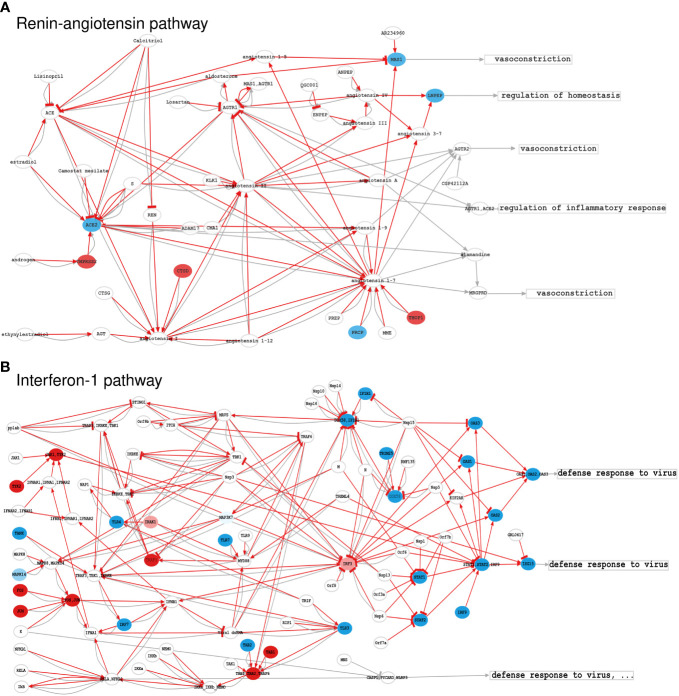
Activation levels of significant C19DMap pathways in SARS-CoV-2-infected nasopharyngeal tissue; **(A)** Renin-Angiotensin pathway, and **(B)** Interferon-1 pathway. Activation levels were calculated using GSE152075 transcriptional data and the HiPathia mechanistic pathway analysis algorithm. Nodes represent genes (ellipses), metabolites/non-gene elements (circles), or functions (rectangles). Pathway-derived circuits connect receptor genes/metabolites to effector genes/functions, simplifying functional interactions into inhibitions or activations. Red arrows indicate circuits activated in infected cells. Node colours correspond to differential expression levels in SARS-CoV-2-infected vs. normal lung cells. Blue: down-regulated elements, red: upregulated elements, white: elements not differentially expressed. HiPathia calculates the overall circuit activation and can indicate deregulated interactions even if interacting elements are not individually differentially expressed.

As thoroughly described in the scientific literature, impaired coagulation is one of the main complications of severe COVID-19, leading to thrombosis and microthrombosis episodes (28). The C19DMap Renin-angiotensin pathway (**Figure 4A**) was converted into 12 circuits, with only one circuit being differentially activated in infected cells. This circuit involves ACE2, widely associated with SARS-CoV-2 infection (29), and its upregulated effector gene MAS1. The upregulation of the MAS1 circuit is related to the normal vascular system functioning (30), and the activation of this axis may result from a vasoprotective response of the glycoproteins, such as GPVI and vWF, involved in thromboembolism, thromboinflammation, and other coagulopathies (31). Hyperactivated platelets in COVID-19 show reduced glycoprotein VI (GPVI) reactivity (32), consistent with our modelling results. The C19DMap Interferon-1 pathway was highly activated, an expected response to virus infection (**Figure 4B**).

### 2.2 Dynamical modelling of host-pathogen interactions on a molecular, cellular, and multicellular level

Next, we studied the impact of upstream regulators on the functional outcome of pathways using dynamic computational modelling, focusing primarily on the Interferon-1 pathway in two different contexts: a pathway and cellular context integrated into a macrophage model. We then integrated the macrophage model into a multicellular context, including the SARS-CoV-2-induced respiratory epithelium’s apoptosis and the virus’s influence on the recruitment of immune cells by macrophages. The tool CaSQ (22) was used to convert the mechanistic C19DMap diagrams into Boolean models (**Figure 4**).

#### 2.2.1 A dynamic Boolean model of type 1 IFN responses in SARS-CoV-2 infection

Type 1 Interferon (IFN) signalling is an essential pathway of host defence against viral attacks, and the corresponding C19DMap pathway was shown to be significantly enriched/activated in all analyses of cell lines and patient sample omics data. We used the tool CaSQ and a previously described map-to-model translation framework (33) to obtain an executable, dynamic Boolean model of type 1 IFN signalling. The dynamic model contained 121 nodes, including three drugs, namely 3,4-methylenedioxy-β-nitrostyrene (MNS) (34), azithromycin (35), and GRL0617 (36) that were included in the diagram by the curators (**Supplementary Figure S7**). We performed simulations for seven scenarios derived from the scientific literature to evaluate the model’s ability to reproduce established biological behaviour (**Supplementary Table S6**).

The sensitivity analyses were conducted based on partial correlation coefficients using Cell Collective (37). The model could reproduce the behaviour for five observations, partially reproduce the behaviour for one, and fail to reproduce one biological scenario (**Supplementary Table S6**, **Supplementary Figure S8**). Global environmental sensitivity analysis results suggest that viral E protein has the highest impact on the inflammation phenotype in the presence or absence of the drugs. Nsp3 shows a negative association with the body’s antiviral response. Sensitivity analysis results in the presence of drugs show that treatment with MNS could reduce inflammation, while azithromycin increases the antiviral response (**Supplementary Figure S9**). Sensitivity analysis against knockout and overexpression perturbations suggests that the overexpression of the IFNB1 RNA has a significant role in the inflammatory process by activating the AP-1 and p50_p65 complexes. The IFNB1 RNA increases pro-inflammatory cytokines by activating the NLRP3 inflammasome, and MNS selectively inhibits it (34, 38). However, overexpression of p50_p65 stimulates the inflammatory cytokines via nuclear reactions regardless of the NLRP3 inflammasome inhibition. Therefore, MNS may need to be combined with other drugs to reduce the inflammation from nuclear reactions. The viral dsRNA and proteins (Nsp13, Nsp14, and Nsp15) can be significant drug targets since they have potent antagonistic interferon effects. TLR7/9 and TREML4 are the most significant viral binding proteins, suggesting TLR antagonists may be used to control exaggerated inflammations via the MYD88_TRAM complex.

#### 2.2.2 Calculating stable states of the IFN model

We used input propagation (39, 40) and control nodes to regroup the model’s inputs and simplify the analysis. We regrouped inputs into six categories: 3 meta-inputs that correspond to Inflammatory stimulus, IFN response, and viral stimulus, and three inputs representing the drugs present in the model (GRL0617, Azithromycin, and MNS). We could identify 128 stable states and no oscillations using this modified model. All signatures lack IFN secretion and exhibit either viral replication or antiviral response. To investigate the model’s behaviour further, we selected eight configurations for the inputs that cover different biological scenarios of the type 1 IFN pathway with or without infection and in the presence or absence of drugs (**Table 2**). We then clustered the stable states according to the four outputs of interest: viral replication, antiviral response, inflammation, and secretion of IFNA1. We have a single attractor for each selected input condition (after projection on the outputs; **Table 2**).

**Table 2:** Input configurations that cover eight different biological scenarios of the type 1 IFN pathway with or without infection and in the presence or absence of drugs.

Input configurations	C1	C2	C3	C4	C5	C6	C7	C8
Viral components	1	1	0	1	1	1	1	1
Immune response	0	0	1	1	1	1	1	1
IFN secretion	1	1	1	1	0	1	1	1
Azithromycin	1	0	0	1	0	0	0	0
GRL0617	1	0	0	0	0	0	1	0
MNS	1	0	0	0	0	0	0	1
Projection of the stable states to the four outputs	C1	C2	C3	C4	C5	C6	C7	C8
ISG_expression_antiviral_response_phenotype	1	0	1	1	0	0	0	0
Viral_replication_phenotype	1	1	0	1	1	1	1	1
Proinflammatory_cytokine_expression_Inflammation	0	1	0	1	1	1	1	0
type_I_IFN_response_phenotype	0	0	0	0	0	0	0	0

The results of the stable state analyses corroborate the results of experimental studies in patients with COVID-19 with various degrees of severity that showed hampered IFN-I responses in patients with severe or critical COVID-19 (41). These patients had low IFN-I and ISGs and increased tumour necrosis factor (TNF-), IL-6-, and NFkB-mediated inflammation. The results of input propagation can be visualised in a heatmap where columns represent all 121 components of the system and rows represent the eight selected input conditions (**Supplementary Figure S10**).

#### 2.2.3 Integration of the Type I IFN, the RA system, and the NLRP inflammasome curated pathways into a macrophage-specific Boolean model

**Figure 5:**
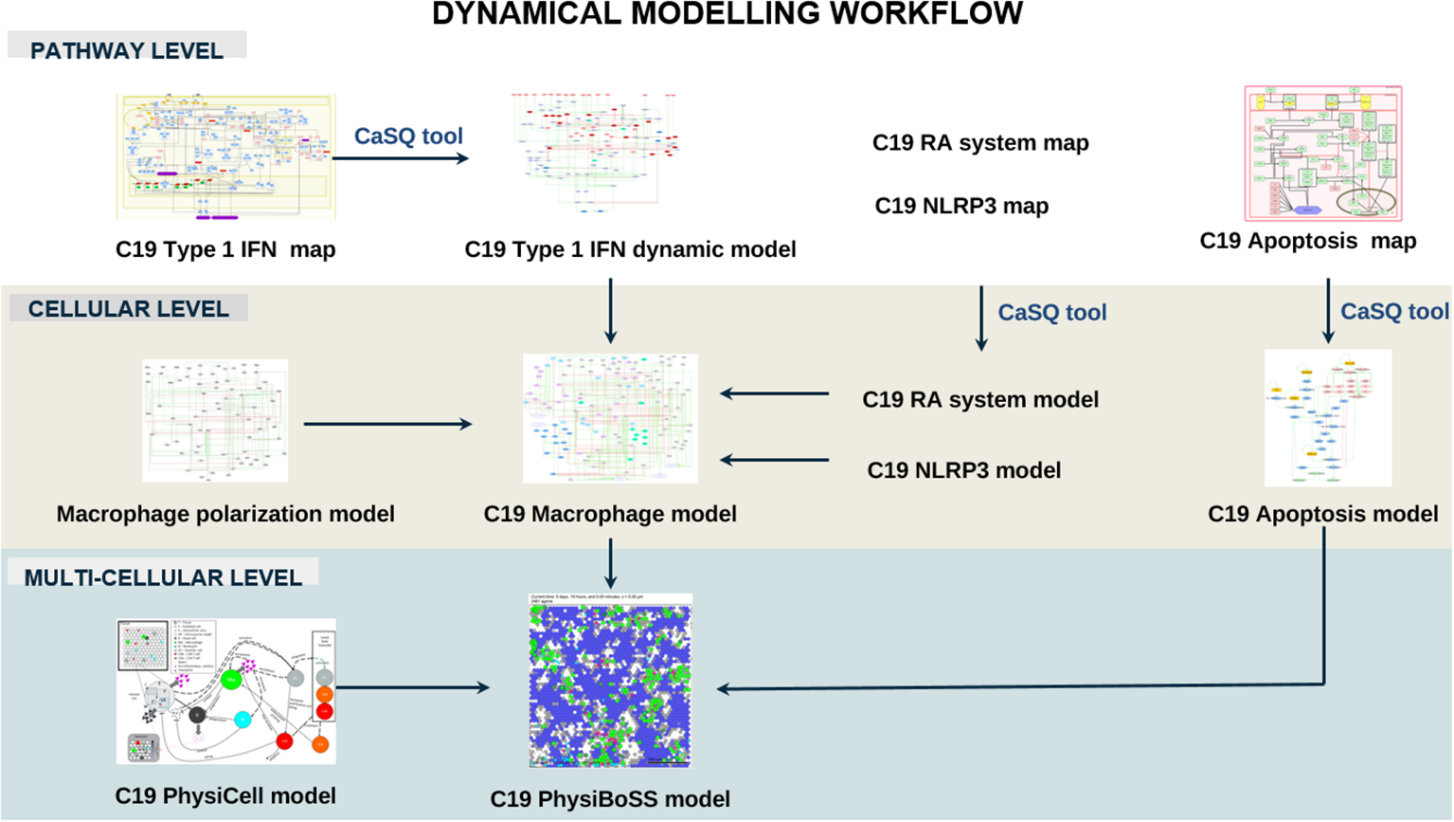
Dynamical modelling workflow of the C19DMap pathways. In this section, we include pathway-level modelling, focused on the Type 1 IFN pathway of the C19DMap, cellular-level modelling, focusing on macrophages, and multicellular level modelling combining macrophages, T-cells and epithelial cells in an Agent-Based Model.

**Figure 6:**
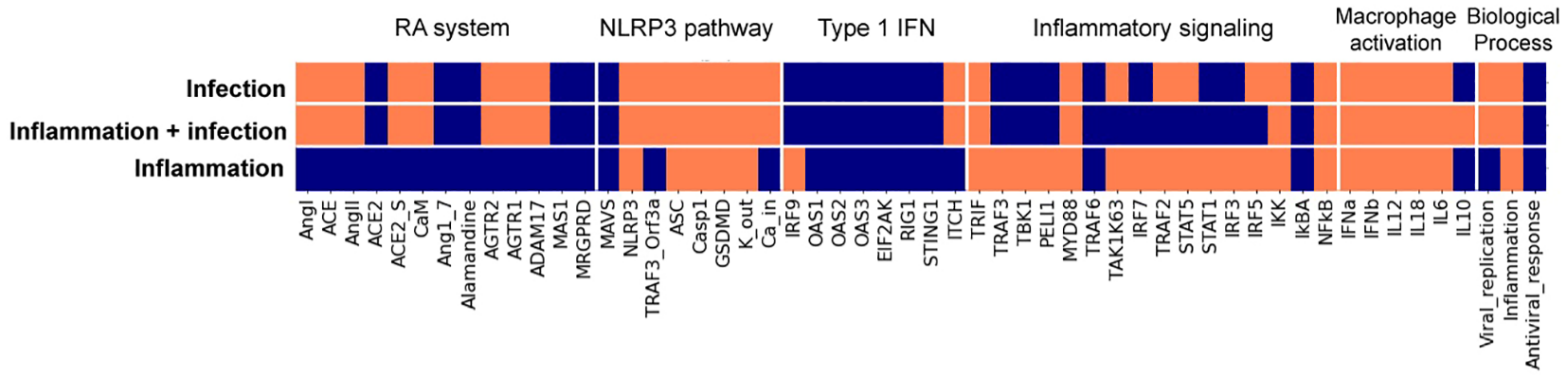
Stable states from the macrophage Boolean model specific for SARS-CoV-2 infection. Model stable states upon different inputs (virus infection, inflammatory conditions + virus infection, and inflammatory condition) are presented in the heatmap. Each input evolves into a unique stable state (rows, delimited by white horizontal lines), where node activity is shown in orange when active and blue when inactive. Nodes, listed at the bottom of the heatmap, are clustered (delimited with white vertical lines) by their relation with specific modules, with the activation of macrophage phenotypes, or with biological processes.

The next step was integrating the IFN response into a relevant cell model. The population of macrophages expands during SARS-CoV-2 infection, and hyperactivation of these cells can lead to severe immunopathologies (42). To computationally simulate the effects of SARS-CoV-2 on selected C19DMap pathways in macrophages, we extended a previously built macrophage polarisation model to incorporate Type 1 IFN response, the Renin-Angiotensin (RA) system, and the NLRP3 inflammasome modules from the C19DMap (workflow is presented in **Figure 5**). The resulting COVID19 Macrophage Model, named MacCOV (https://gitlab.lcsb.uni.lu/computational-modelling-and-simulation/macrophage-model), comprises 131 nodes and 271 edges manually verified against the macrophage-specific literature. When an inflammatory microenvironment stimulus is simulated, the model reaches a stable state with the respective signalling cascades and inflammatory biomarkers rendered active (inflammatory response; **Figure 6**). Infection with SARS-CoV-2 stimulates the RA system module, which potentiates inflammation through specific mediators and effectors, like AGTR1/2. Consistent with the literature (43, 44), the virus, through an Orf3a_TRAF3 complex, also triggers the activation of the NLRP3 inflammasome, thus leading to cleavage of proIL-1b and proIL-18 into their functional forms. In addition, although the inflammatory stimuli remain, the stable state analysis indicates that the virus can directly activate the expression of pro-inflammatory markers without activating the central signalling cascades. SARS-CoV-2 itself is sufficient to trigger an inflammatory response in macrophages. The virus can also block the type 1 IFN signalling at different cascade levels, as demonstrated in the molecular-level model. Lastly, the virus also blocks nodes from inflammatory pathways, which crosstalk with the type 1 IFN pathway. By binding to their cognate receptors, pro-inflammatory mediators activate their downstream signalling effectors, which typically converge on a core pathway (i.e. one that captures signalling from other cascades) or a critical pro-inflammatory transcription factor such as NFkB.

#### 2.2.4 Multiscale and multicellular simulations of SARS-CoV-2 infection uncover intervention points to evade respiratory epithelium apoptosis and increase immune cell recruitment.

**Figure 7:**
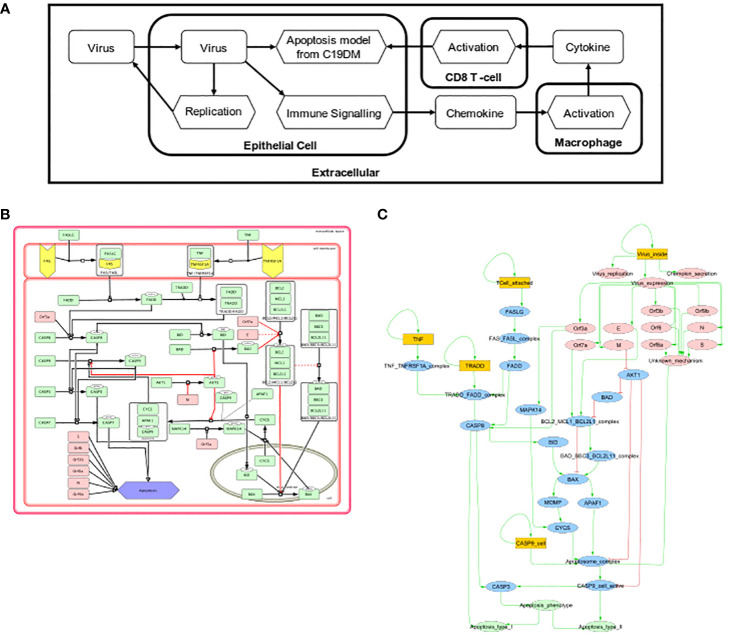
Multiscale simulation workflow. **(A)** Overview of the top-level interaction model that integrates virus infection, host respiratory epithelial cell demise, and the response of different immune cells. **(B)** The apoptosis model from C19DMap (https://fairdomhub.org/models/712). **(C)** The modified version of the apoptosis model was included in each respiratory epithelial cell type.

Two Boolean models focusing on the effects of SARS-CoV-2 on respiratory epithelium apoptosis and the recruitment of immune cells by macrophages were incorporated into a multiscale simulator of the infection of lung epithelium by SARS-CoV-2 (45) [https://git-r3lab.uni.lu/computational-modelling-and-simulation/pb4covid19] (**Figure 7**). CaSQ (33) was used to convert the apoptosis map into a Boolean model.

Models were analysed individually by studying each Boolean model’s knockout (KO) (46) to identify potential drug targets. Two perturbations were identified: one that evades apoptosis in infected human host respiratory epithelial cells and one that increases the immune response in macrophages (**Supplementary Figure S11**). The first perturbation involved the inhibition of FADD, a downstream actuator of FASLG reception upon T-cell activation promoting apoptosis. In the FADD knockout simulation, CD8-T-cell-mediated apoptosis was abrogated, but the cells could still undergo virus-mediated apoptosis through activation of the apoptosome by the virus (**Supplementary Figure S12A**). The second perturbation inhibited the macrophages’ p38, a MAP kinase that phosphorylates various proteins in response to stress. The knockout of p38 in this macrophage model increased the recruitment of immune cells by 10% (**Supplementary Figure S12B**). We studied the population of respiratory epithelial cells and their status (**Supplementary Figure S13A**) and the recruitment of immune cells (**Supplementary Figure S13B**).

The effect of the mutations was incorporated in the multiscale simulation. In the multiscale model, FADD KO behaviour corresponded to the expected behaviours observed in the Boolean model as it reduced the commitment of respiratory epithelial cells to apoptosis (**Supplementary Figure S13C**). In the multiscale model, p38 KO did not substantially change immune cell recruitment by macrophages (**Supplementary Figure S13D**). The 10% increase in the recruitment of immune cells in the signalling model was insufficient to see consistent differences when replicating conditions in the multiscale simulation.

### 2.3 Drug target enrichment and pharmacogenomics analysis

**Figure 8:**
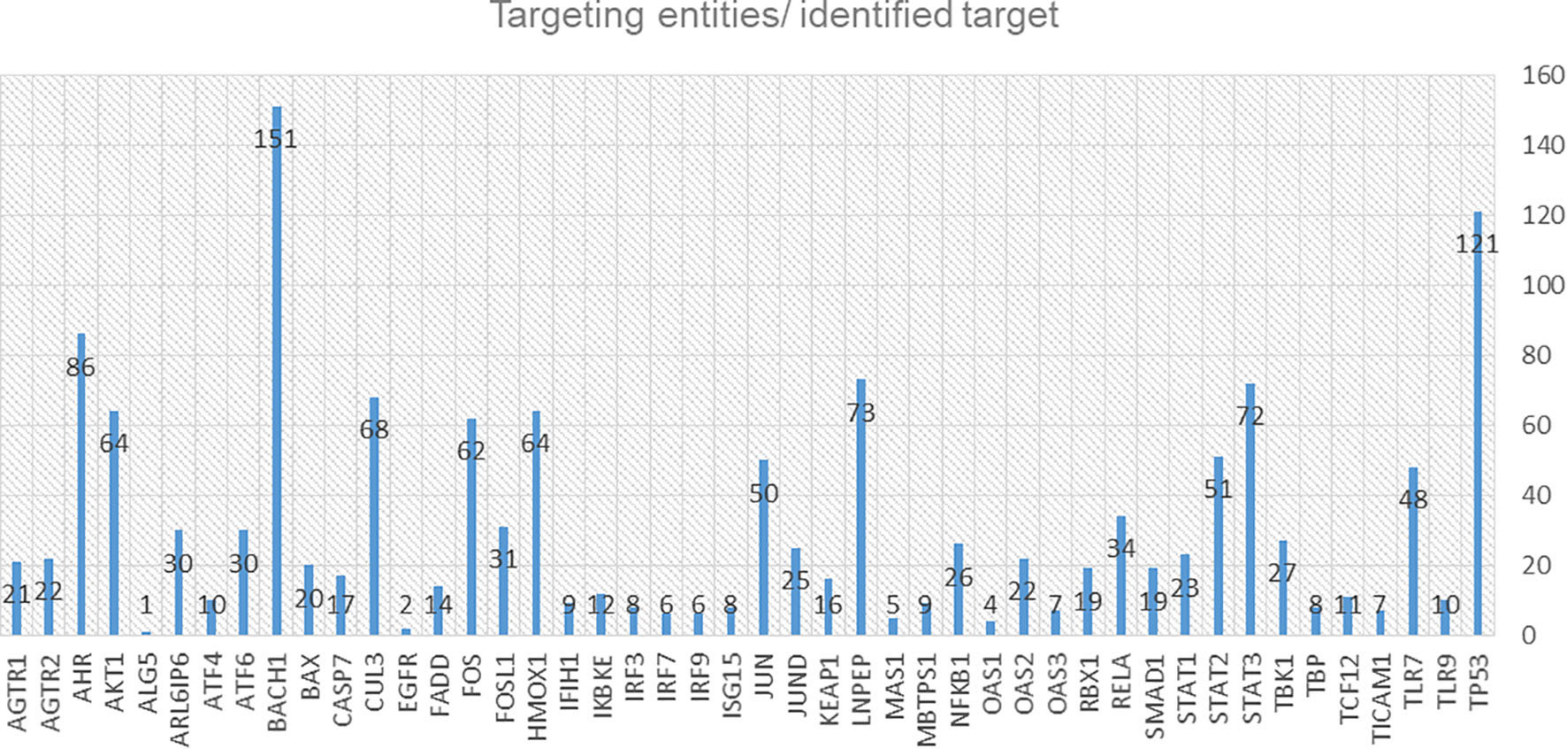
Diagram of the identified targets and the corresponding targeting entities (drugs, chemicals, mirRNAs, small molecules).

We identified 54 targets from the integrative omics data analyses, and the computational modelling is already included in the C19DMap diagrams (**Supplementary Table S7**, **Supplementary Figure S14**). Two AI assistants, INDRA and AILANI, were used to compile a list of drugs and drug targets using the repository’s content and information from various external sources such as Clinical trials DB, Drug Bank (47), ChEBI (48), mirTarBase (49) and scientific literature. From an initial list of 3,573 proteins extracted from the C19DMap and the drug-target information compiled for the C19DMap, we obtained 1,476 drugs associated with 1,120 drug targets to populate our C19DMap drug-target database. Using the C19DMap drug-target database, we inferred 1,429 drugs, chemicals, and miRNAs that target the identified nodes (**Supplementary Table S8**). If we remove viral proteins and focus only on drugs and chemicals, there are 228 unique drugs/chemicals for 46 targets, as shown in **Figure 8**.

**Figure 9:**
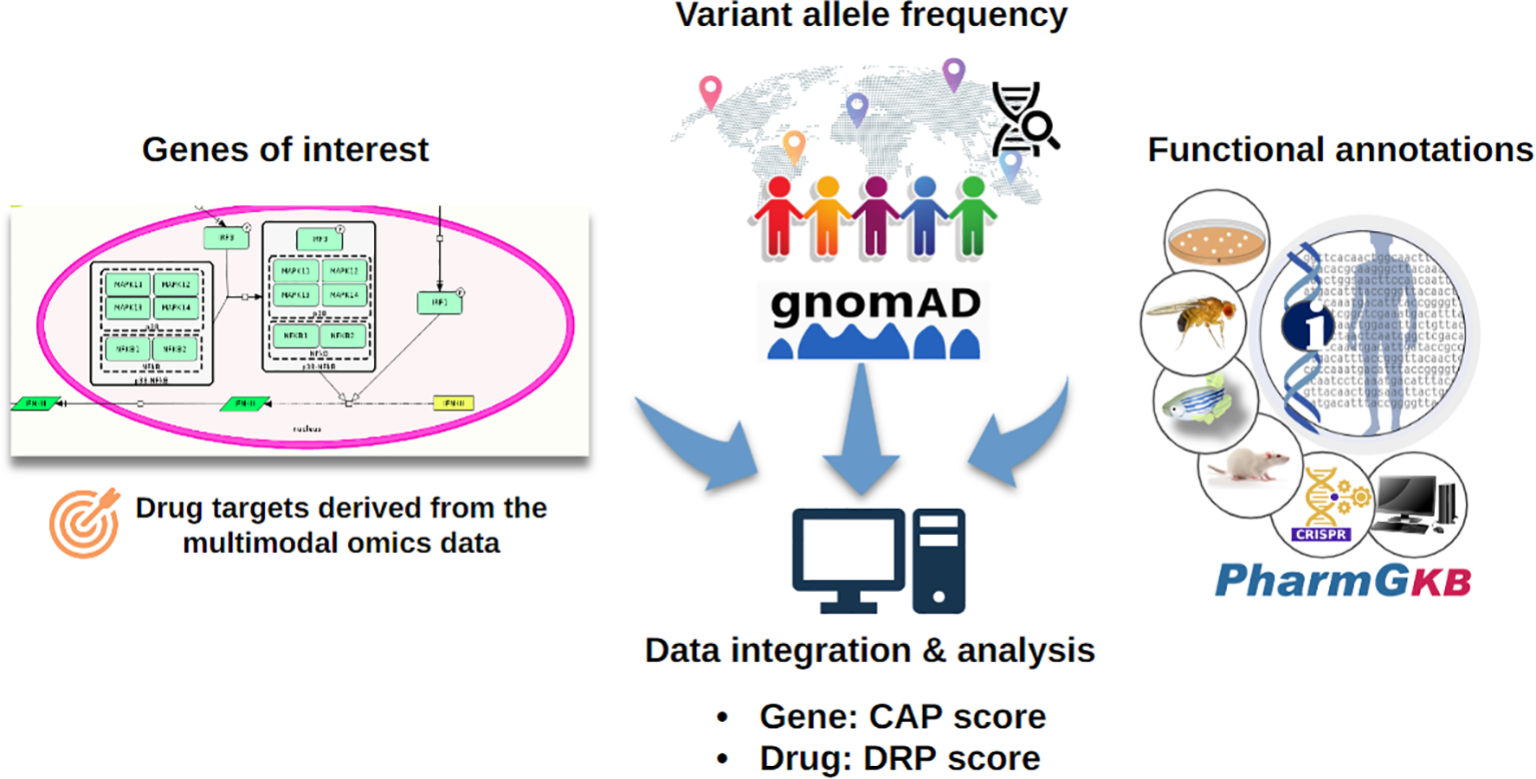
The CAP score estimates the likelihood of a particular gene carrying pharmacogenomic variants, while the DPR score estimates the likelihood of the response to a drug being affected by pharmacogenomic variants (50). The CAP score depends on the number of pharmacogenomic variants and their population frequency.

Pharmacogenomic information for the drug targets in the C19DMap was collected from the public domain, and the frequency of these genomic variants was assessed. The “Cumulative Allele Probability” (CAP) and the “Drug Risk Probability” (DPR) scores were used to summarise the data (**Figure 9**). We focused on 79 genes with available pharmacogenomic information and allelic frequency data in PharmGKB and gnomAD, respectively, and (**Supplementary Table S9**) calculated CAP scores using gnomAD global exomic information (**Supplementary Figure S15**). The individual CAP scores for the drug target genes were aggregated by drug (**Supplementary Figure S16**).

Losartan, an antagonist of the angiotensin II receptor, type 1 (AGTR1), is used for hypertension treatment (51). Two AGTR1 genomic variants to losartan response are annotated in PharmGKB (rs5186 and rs12721226). The variant rs5186 was associated with an increased response to losartan in a cohort of European ancestry (52). The other variant, rs12721226, a missense variant with very low frequency across populations, is associated with a decreased affinity to losartan, which could impair the drug’s clinical efficacy (53). INDRA and AILANI analysis retrieved additional drugs and miRNAs, besides losartan, able to target AGTR1. Pharmacogenomics data was also available for identified IKBKE, CASP7, and EGFR targets. For IKBKE, the CAP score is very low across all populations. INDRA analysis retrieved many chemicals and two drugs, amlexanox and sunitinib malate, that target IKBKE, while AILANI analysis retrieved the miRNAs hsa-miR-124-3p, hsa-miR-155-5p and hsa-miR-296-5p (**Supplementary Figure S16**). Amlexanox, used in four clinical trials targeting type 2 diabetes and obesity, has no pharmacogenomic data. Regarding CASP7, the CAP score is very high for East Asians, both male and female and very low for African/African American populations. INDRA analysis retrieved spermine, 1,4-benzoquinone, melatonin, apigenin, zinc, cisplatin, ac-asp-glu-val-asp-h, NAC, fica and emricasan, while AILANI analyses retrieved eight miRNAs that can target CASP7. Pharmacogenomic data were only available for cisplatin. Cisplatin has a higher DRP score for Latino/Admixed Americans, both sexes, and a lower DRP score across Ashkenazi Jewish and East Asian populations (**Supplementary Figure S16**). Emricasan was tested in 18 clinical trials targeting liver diseases and has recently been tested for its efficacy in COVID-19 in 13 patients with mild symptoms, but no results have been published^3^

Lastly, EGFR’s CAP score is very low across all populations slightly higher for African/African American populations. Using our internal drug-drug target database, we retrieved two drugs: zanubrutinib and abivertinib. Zanubrutinib is being tested in clinical trials for treating lymphoma (88 clinical trials in ClinicalTrials.gov). Abivertinib has been tested in 11 clinical trials for lymphoma, prostate, and lung cancers and recently evaluated in two completed clinical trials for COVID-19, according to ClinicalTrials.gov.

### 2.4 AI-assisted map updating and expanding

The multimodal data analysis described in this work highlighted new molecules and pathways, with key functions regarding the progression of the SARS-CoV-2 infection, not yet annotated and wired in the C19DMap repository. We now have an extensive list of TFs, pathways and DEGs identified as active in SARS-COV-2 infection that would need to be incorporated into a 2nd-generation C19DMap. We use two text mining and AI-assistants to keep the context up to date and further expand and enrich it with new knowledge. One of the major problems in wiring new molecules in the diagrams is the need for mechanistic details. AILANI and INDRA (Integrated Network and Dynamical Reasoning Assembler) were used to infer interactions to link new molecules into the diagrams.

The AILANI COVID-19 research assistant (https://www.labvantage-biomax.com/products/ailani-for-semantic-integration-and-search-2/) is based on a previously developed natural language processing and machine learning-based text mining pipeline (54) and a novel artificial intelligence-based question-answering system. The AILANI assistant continuously mines Medline abstracts, public PubMedCentral full-text articles, COVID-19 specific collections from bioRxiv/medRxiv, Elsevier, and the Allen Institute for AI COVID-19 (CORD-19), ClinicalTrials.gov and relevant newsfeeds (e.g., WHO, CDC, NIH). The AI is based on deep neural networks trained to identify objects and, therefore, can provide novel insights and associations that are not (yet) part of explicit semantic networks. We also used INDRA (4), an open-source automated knowledge assembly system integrating information from published literature and biological pathway databases to enrich the C19DMap diagrams. We systematically aligned the C19DMap with assembled INDRA Statements to enrich (i.e., find additional literature evidence for interactions already incorporated) and extend (i.e., find relevant interactions that have not yet been incorporated) the C19DMap. We provide two small examples that showcase how new interactions and biomolecules can be integrated into the repository. **Table 3** provides an example of new interactions for molecules already included in the C19DMap repository, while the second example describes the wiring of newly identified TFs.

**Table 3:** Example of new interactions between pathways and within the same pathway for a given node, inferred using the two AI assistants, AILANI and INDRA.

Identified node in the C19DMap repository	Submaps, including the node in C19DMap	Identified interactor Uniprot ID	Identified interactor HGNC	AI-assistant	Submaps of the C19DMap, including the identified interactor	Type of information
MAS1	Coagulation pathway; Renin-Angiotensin	P05231	IL6	INDRA (EMMAA)	Coagulation pathway; Interferon lambda pathway; Nsp14 and metabolism	Link between pathways, new interaction within the same pathway
MAS1	Coagulation pathway; Renin-Angiotensin	Q14116	IL18	INDRA (EMMAA)	NLRP3 inflammasome activation; HMOX1 pathway	Link between pathways
MAS1	Coagulation pathway; Renin-Angiotensin	Q9UI12	ATP6V1H	AILANI	Nsp4 and Nsp6 protein interactions	Link between pathways

Regarding information about new TFs and DEGs highlighted from the multi-omics data analysis, AI assistants can provide information for their wiring through direct and indirect links. For example, TF activity analysis revealed a set of TFs common for A549 and NHBE cells (**Supplementary Table S2**). KLF6 was among the TFs not yet present in the C19DMap repository. The AI-assisted analysis revealed two possible interactions with CDKN1A and PDFGA. CDKN1A and PDFGA are not yet included in the C19DMap database; however, both molecules have been identified as potential interactors for numerous biomolecules in the repository, providing an indirect way of wiring the TF- interactor pairs. Moreover, CDKN1A is present in the T-cell activation SARS-CoV-2 (Homo sapiens) WP5098, which will be included in the pathway collection in the next update scheduled for March 2024.

### 2.5 Graphical exploration and topological analysis

To cope with the size and complexity of the ever-growing content of the mechanistic pathways, we developed and implemented a concept for the hierarchical exploration of the C19DMap and performed a comprehensive analysis of node centralities on two levels: individual pathways level for all three platforms (MINERVA, WikiPathways, and Reactome) and on the level of an aggregated network combining all individual pathways. The implementation is based on the biological network analysis tools Vanted (55), SBGN-ED (56) and LMME-DM, a customised version of LMME (Large Metabolic Model Explorer) (57) (**Figure 8**).

The centrality analysis was performed on all networks combined in a bipartite graph (individual pathways and aggregated network). An aggregated centrality value was computed (see Materials and Methods) to identify the top-ranked instances of the C19DMap bipartite graph (**Supplementary Table S10**) from a topological perspective. Not surprisingly, the top ten proteins were viral proteins and ACE2 that act as a receptor for the SARS-CoV-2 spike protein.

**Figure 10:**
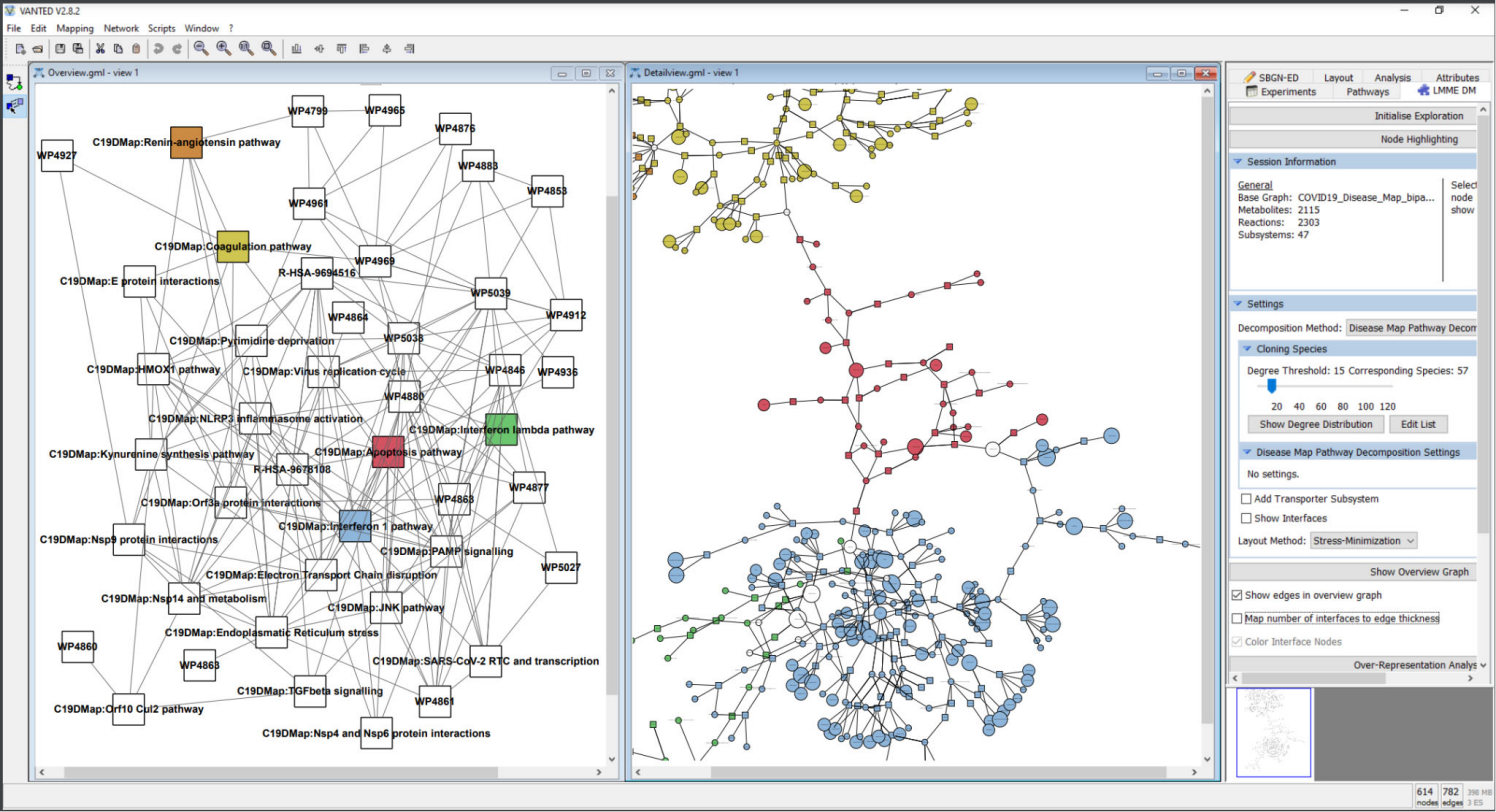
Hierarchical exploration of centrality values in the disease map using LMME-DM. The following pathways are detailed: Coagulation: yellow; Apoptosis: red; Interferon 1: blue; Interferon lambda: green; Renin-Angiotensin: orange. The aggregated centrality values are mapped to the node sizes in the detail view.

Topological analyses can highlight targets and hubs, providing a basis for linking pathway structure with key findings from text mining, omic data analysis, and modelling pipelines. For the five representative C19DMap pathways, namely Itype 1 IFN, Interferon lambda, coagulation, apoptosis, and renin-angiotensin, we used the aggregated ranks to create a high-level view of the pathways, visualising their connections and also creating nested nodes to handle complexity (**Figure 10**). Of the 54 highlighted targets (**Supplementary Table S7**), nine are characterised as structurally important in the respective pathways, namely TBK1, IKBKE, IRF3, MAS1, IRFNB, CASP7, FADD, AKT1, and AGTR1/2, as they appear in the top ten instances of the five individual pathways in the C19DMap bipartite graph (**Supplementary Tables S11–S15**). The topological features for the aggregated network (unified content across the three platforms, MINERVA, WikiPathways, and Reactome) were not always easy to calculate due to incompatibilities that will be addressed in the future versions of the repository (e.g. different naming for the same protein complex, such as AP-1 or AP1, different spelling or capitalization of node names, such as nsp13 or Nsp13). Clean topological features were available for 26 of the 54 targets. Among these, 11 targets were considered structurally important as they were in the top 30% of instances of the aggregated network by aggregated centrality values (**Supplementary Table S16**). The minor inconsistencies in the data, for example, different names for the same molecule or the use of names of complexes instead of names of the complex components, as showcased in the examples above, were resolved using the UniProt IDs. There are a few cases where this was not possible. However, to be consistent with the dataset used in other analysis steps, we did not try to resolve these cases before the topological analysis. The main findings of the topological analysis are not impacted.

### 2.6 FAIRness and availability for proper data management

An ongoing effort is aligning our work with the four FAIR principles: Findability, Accessibility, Interoperability, and Reusability (58).

Findability: Our resources are publicly available via our git and dedicated repositories. The tools implemented in our ecosystem are published and indexed on PubMed and searchable online. We invest efforts in advancing, communicating and exchanging with other Systems Biology communities, especially regarding the annotation and curation of models (59, 60). Furthermore, besides providing the source files, we will also make the models obtained available in various model repositories, such as the Cell Collective (37), GINsim (61), and BioModels (62), with the publication of this manuscript. Appropriate metadata associated with each of the analyses and modelling results presented in the article is registered and indexed on FairdomHub to facilitate discovery.

Accessibility: All tools are open access except for AILANI (54) which requires registration. WikiPathways (22), REACTOME (23), MINERVA (63), INDRA (4), and CellCollective (37) also provide open-access APIs. The developed maps and models are available on GitLab (https://git-r3lab.uni.lu/covid/models/) and FAIRDOMHub (64).

Interoperability: We have worked on tool interoperability and promoting community standards; therefore, most input formats are GML, SIF or SBML, and SBML Qual files to enhance model reusability (65).

Reusability: All maps and models are available under a CC-BY licence.

We have also built the C19DMap-Neo4j graph database by integrating the content of the C19DMap diagrams available in MINERVA into the Neo4j framework. This database is available for online exploration at https://c19dm-neo4j.lcsb.uni.lu and is used as a backend solution for efficient access to the resource data. Biological concepts from the C19DMap diagrams available in MINERVA (such as macromolecules and processes) are stored in the database under Neo4j nodes. In contrast, relationships between these concepts (such as consumption and catalysis) are stored as Neo4j relationships. In addition, annotations, such as UniProt identifiers and PubMed publication IDs, are stored as individual nodes that we can easily query (for example, see **Supplementary Figure S17**).

## 3 Discussion

We have explored the high-quality, manually curated mechanistic content of host-pathogen interactions of the C19Dmap using several bioinformatics analytic and computational systems biology tools that are now combined in interoperable pipelines. To further prioritise targets and contextualise the mechanistic content with different layers of biological data, a set of different omics data was used, ranging from infected cell lines to bulk RNAseq and single-cell omic data from patients affected with SARS-CoV-2. In summary, we used omics data following SARS-CoV-2 infection to infer a causal network describing signalling events perturbed after viral infection. We identified the MAPK protein family as a critical mediator of the referred signalling events. Our omics-based approach captured several genes in the pathways manually curated by the C19DMap community.

Furthermore, we found additional causal interactions suggesting the potential mechanism behind the crosstalk between some of the most relevant pathways upon SARS-CoV-2 infection, such as EGFR, PI3K, and the PAMPs/interferon-1 pathway. Regarding transcription factors, the analysis revealed new transcription factors not yet included in the C19DMap. Their inclusion may provide an opportunity to reveal more detailed mechanisms of gene regulation hijacked by coronavirus infection. The results showed that, among the drugs targeting transcription factors detected in both cells, 47 were already in external clinical trials, including drugs evaluated for their effectiveness against COVID-19. In addition, we also retrieved 160 drugs that have not yet been tested in clinical trials or tested for efficacy against COVID-19 and could represent potential candidates for further evaluation (**Supplementary Table S17**). Lastly, the over-representation analysis revealed 58 affected pathways in NHBE cells and 39 enriched pathways in A549 cells, including pathways relevant to immune response, the NFkB pathway, glucocorticoid receptor and MAPK signalling pathways, and pathways related to interferon.

The single-cell RNAseq data analysis of a small group of patients confirmed some of the previously identified TFs, DEGs and altered pathways the cell line analysis pointed out. However, the number of patients in this analysis was relatively small. To expand our analysis, we used an extensive dataset of 450 patients and the HiPathia modelling algorithm to identify affected circuits in the mechanisms described in the repository. Mechanistic models of signalling pathways provide a conceptual framework for interpreting gene expression or genomic variation data. These methods have been developed to associate gene activity with their consequences over downstream processes and phenotypic responses, which are highly relevant for studying disease progression or drug response, especially in complex diseases.We found pathways, such as apoptosis, to be systematically up or downregulated, which means that the whole pathway is relevant to the progression of the disease. Moreover, more extensive pathways showed differential activation in a few or even one of the circuits, which may indicate that, despite the involvement of the whole pathway in the disease progression, only a few processes reflected in the deregulated circuits are critical to the mechanism of infection. These specific key processes may support finding new therapeutic targets.

The extensive integrative omic data analysis using bulk RNA-seq, scRNA-seq and the pathway resources revealed interesting TFs, DEGs, and altered pathways after the SARS-CoV-2 infection in the two studied cell lines and patient data. The methodologies used for this step were complementary, covering a wide range of state-of-the-art pipelines and bringing forward two significant points: the coverage and relevance of the C19DMap repository regarding the COVID-19 disease and identifying additional regulators that would need to be included in the resource.

The C19DMap can also be analysed using computational modelling approaches to help elucidate mechanisms deregulated at molecular, cellular, and multicellular levels, thus gaining insight into COVID-19’s underlying processes. Type 1 IFN signalling is an essential pathway of host defence against viral attacks, as highlighted in previous omics data analyses in cell lines and patients’ samples. We used our repository’s executable, dynamic model of type 1 IFN signalling for in-silico experimentation. The computational modelling results showed a complete lack of IFN signatures under relevant conditions matching the experimental results that showed hampered IFN-I responses in patients with severe or critical COVID-19 (36). These patients had low IFN-I and ISGs and increased TNF-, IL-6-, and NF-κB-mediated inflammation. Adding the IFN response, Renin-Angiotensin mechanism, and NLRP3 pathways from the C19DMap to an existing macrophage polarisation model helped elucidate the innate immune response that macrophages trigger upon acute COVID-19, in addition to highlighting their contribution to the disease’s pathology. Lastly, integrating both pathway and cell models in a multicellular-multiscale model helped reveal the impact of mutations of FADD and p38 on the cellular death of epithelial cells upon infection and the recruitment of immune cells. Whereas these results demonstrate the value of COVID-19 disease logical models for generating new hypotheses and understanding of disease mechanisms, they also provide an important tool set for preclinical discovery and testing of targeted drugs and drug combinations, as demonstrated in several studies (7, 66–68). In-silico model simulations can prescreen many drug combinations, with the best-performing candidates advancing to further preclinical testing. *In vitro* validation of the models’ prediction can accelerate preclinical testing by narrowing down the number of candidates and combinations, improve the performance of computational models by addressing failures, and guide experimental design.

To expand the content, AI-assisted text mining systems, such as INDRA and AILANI, were employed to infer from the vast literature the drugs, miRNAs and chemicals that target biomolecules included in the diagrams of the C19DMap. Besides expanding the content, text mining and AI solutions provide directions to fill knowledge gaps. Furthermore, integrating publicly available data from the C19DMap, PharmGKB, and gnomAD allowed us to determine the presence of variants with pharmacogenomic impact and their frequency in human populations. We thus estimated the genomic variability of genes from the C19DMap involved in drug response across different populations and sexes. We retrieved pharmacogenomic information for about 79 genes in the repository, four of which were identified as potential targets. Topological analyses revealed important information about hubs and shared molecules among pathways that could help us better understand the potential upstream and downstream effects of targeting them. We are aware of minor inconsistencies in the unified database, for example, name variations for the same molecule or the use of names of complexes instead of names of the components of the complex. While the main findings of the topological analysis remain unaffected, we aim to harmonize the content as much as possible during the following repository content updating. The C19DMap project is an ongoing effort, and our goal is to maintain the repository and keep it updated with improved content.

It is important to note, that besides the inconsistencies regarding the diagrammatic content of the repository, the omics data integration and modelling approaches come also with certain limitations. Regarding data analysis and preprocessing, we used the same package and preprocessing steps to obtain results as harmonized as possible. However, we have used data from various sources and while the main findings do not change, some TFs or DEGs might be context specific. Our logic-based modelling approaches may oversimplify the interactions between molecular entities. This simplification could potentially obscure some insights that the data might otherwise reveal, particularly in the complex interplay of different biological pathways. As our models are qualitative, we lack ways to address quantitative questions, especially in the context of drug simulations. Additionally, while our transcriptomic analysis serves as a proxy for protein activity, we acknowledge that overexpression of genes does not directly equate to active protein function. Such limitations in our methods should be considered when interpreting the findings and may point to the need for more nuanced approaches in future research to fully understand the mechanisms at play in the progression of the disease.

As mentioned in our previous report (1), most of the diagrams of the CD19DMap repository were initially built using the scientific literature on SARS-CoV-1 and other coronaviruses available during the onset of the pandemic. This corpus provided the foundation for rapid curation and a literature triage approach. Annotations for the SARS-CoV-1 viral infection process, including the viral life cycle, host interactions, and therapeutic pathways, were built on this foundation. After more than two and a half years since the appearance of the SARS-CoV-2 virus, the body of scientific literature specific to this type of coronavirus has reached a point where it can now be used to curate complete mechanisms. With the continuous update of pathway information and new datasets related to SARS-CoV-2, reproducible and automated data analysis workflows can be rerun to provide more accuracy and specificity. Generation of Reactome’s SARS-CoV-2 pathway leveraged the database’s foundational manual curation, orthoinference projection, and the collaborative resources of the C19DMap project. The SARS-CoV-2 infection pathway emerged from a computationally generated rough draft via orthoinference from the manually curated, peer-reviewed Reactome SARS-CoV-1 infection pathway (see Materials and Methods). The community can adopt this approach that identifies SARS-CoV-2-specific interactions to increase viral specificity in the mechanisms included in the C19DMap repository.

## 4 Conclusions

We made considerable efforts to increase interoperability and communication across three different platforms, MINERVA, WikiPathways, and Reactome, support Systems Biology standards such as SBGN (69) and SBML (3), and promote scientific openness with the use of public repositories and the adoption of FAIR (Findability, Accessibility, Interoperability, and Reusability) Data principles (58).

We have successfully built workflows to use high-quality, curated mechanistic content for integrative analysis and computational modelling. The interoperable pipelines developed and demonstrated here are highly adaptable to new challenges due to standardised formats, can support the testing of combinatorial therapies, as multiple drugs and targets are suggested, and offer a canvas for evaluating the repurposing of existing drugs to fight new waves of COVID-19 or other pandemics, and contribute to elucidating the etiologies of post-acute Covid Symptoms (PASC).

By comparing the mechanisms and drug targets, we can further look into the comorbidities of the disease. Moreover, our approaches directly apply to other pathologies, for which mechanistic content and omics data analyses can be combined to identify new druggable points. This combinatorial approach is helpful for rare diseases, where the data is scarce and integrative methodologies can help fill the data gaps.

All pipelines, workflows, tools, and methodologies that comprise the C19DMap computational framework are freely available to the scientific community (See Material and Methods section, and Data availability statement). While we acknowledge the complexity of the C19DMap ecosystem, which stems from the plurality of resources, we remain committed to improving interoperability and standards to facilitate integrated, start-to-end bioinformatics and modelling analyses. We aim to help leverage technological and methodological advancements and lower the accessibility barrier for several tools, methods and approaches that otherwise would remain far off reach for a substantial number of end-users.

## 5 Materials and methods

### 5.1 Using the mechanistic diagrams for omics data analysis

#### 5.1.1 Footprint analysis

We obtained the transcriptomics dataset from the GEO database with accession number GSE147507 (12). We extracted series five from the dataset, consisting of 2 conditions: A549 cells either mock-treated or infected with SARS-CoV-2, measured in triplicate 24 hours after infection. Differential analysis of the transcript abundances was performed using DESeq2 (70). The resulting t-values of the differential analysis were used as inputs to estimate pathway activity deregulation using Progeny (71). The differential analysis t-values were also used to estimate the deregulation of TF activities using Dorothea (72) as a source of TF-target regulon and the Viper algorithm (73) to estimate the TF activity score. Phosphoproteomic data of mock-treated and SARS-CoV-2 infected cells were extracted from (13). Phosphosite differential analysis log2FC was used to estimate the deregulation of kinase activities using https://github.com/indralab/protmapper as a source of kinase-substrate interactions and a z-test to estimate kinase activity score (74, 75). Finally, we used Carnival (15) with the COSMOS approach (16) to connect the top 10 deregulated kinases with the top 30 deregulated TFs with a Prior Knowledge Network assembled from OmniPath resources (11). Progeny pathway activity scores were used to weigh the PKN and facilitate the optimal network search to connect kinases and TFs. To place our results in the context of the whole study, we matched the genes obtained in carnival results with those included in the curated pathways by the C19DMap community (https://covid.pages.uni.lu/map_contents). In addition, we matched our results with a harmonised list containing drug targets. All code and analysis are available here: https://gitlab.lcsb.uni.lu/computational-modelling-and-simulation/footprint-based-analysis-and-causal-network-contextualisation-in-sars-cov-2-infected-a549-cell-line.

#### 5.1.2 TF activity and drug target identification

This analysis inferred the gene regulatory systems hijacked by COVID-19, especially the target transcription factors. In order to infer the target transcription factors, we detected transcription factors that statistically significantly regulate the genes whose expression changes were induced by COVID-19. First, the gene groups whose expression changes were induced by COVID-19 in NHBE cells and A549 cells were detected as the DEGs using DESeq2 (70) for the GSE147507 dataset (12, 14), described above (DEGs; adjusted p-value < 0.05). Next, we extracted all the regulatory relationships with Confidence “A”, “B”, and “C” from DoRothEA (72) as information on the regulatory relationships of transcription factors to each of these DEGs for NHBE cells and A549 cells. The transcription factors that regulated each of these DEGs for NHBE cells and A549 cells were detected by LAMP (10) (significance level < 0.05). Next, to gain insight into the biological phenomena affected by the detected transcription factors, i.e. the transcription factors hijacked by COVID-19, gene ontology enrichment analysis of DEGs under the control of these transcription factors was performed using the GOstats package (76) in R (significance level α = 0.05). In order to verify whether these transcription factors are included in the publicly available C19DMap (1), we performed a search based on the HGNC ID of each transcription factor against the SBML file of each Disease Map. Finally, we searched for and picked up the drugs targeting each transcription factor for NHBE cells and A549 cells in the clinical trials in anticipation of their later usefulness in treating COVID-19. To find the drugs targeting the above transcription factors, we searched against GeneCards (https://www.genecards.org/) (77) based on the HGNC IDs of the transcription factors. After that, we performed another search based on those drugs against the list of the drugs in External Clinical Trials for COVID-19 and Related Conditions in the COVID-19 Dashboard of DrugBank (https://go.drugbank.com/covid-19) (78). Only approved drugs were listed as candidate drugs in the final results. Finally, to identify gene regulatory systems affected by COVID-19 independent of cell type, DEGs, transcription factors, enriched GO terms, and drug targets detected were classified as NHBE-, A549-specific, or shared to both cell types. All code and analysis are available here: https://gitlab.lcsb.uni.lu/computational-modelling-and-simulation/generegulationanalysis.

#### 5.1.3 Pathway and network analysis in SARS-CoV-2 infected NHBE and A549 cells

We demonstrate an automated and reproducible workflow for transcriptomics data analysis using pathway- and network-based approaches (see our GitLab repository for details; https://gitlab.lcsb.uni.lu/computational-modelling-and-simulation/pathway-analysis-and-extension). The analyses are fully automated in R with clusterProfiler (79) and RCy3 (80) to connect to the widely adopted network analysis software Cytoscape (81) for network visualisation. We obtained the transcriptomics dataset from the GEO database with accession number GSE147507 (12). We extracted series numbers 1 (NHBE) and 5 (A549) from the dataset, consisting of 4 conditions in triplicate, NHBE and A549 cells treated with mock (two controls), and NHBE and A549 infected with SARS-CoV-2, measured 24 hours after infection. Pre-processing and differential gene expression analysis were performed in R using the DESeq2 package (70). Next, a combined pathway collection of the C19D Map [21 pathways (82)], WikiPathways [597 pathways (22)] and Reactome [1,222 pathways (23)] were created. Pathway enrichment analysis was performed using the clusterProfiler R package (79). Differentially expressed genes (DEGs; p-value < 0.05 and absolute fold change > 1.5) were used for the over-representation analysis. The analysis was performed separately for NHBE and A549 cells, and the overlap in enriched pathways was analysed. Selected pathways were visualised in Cytoscape using the WikiPathways app (83). A pathway-gene network for the shared pathways was created to study pathway crosstalk and overlap. Next, the harmonised bipartite graph created a pathway-gene network for all C19DMap pathways. By overlaying information about shared differentially expressed genes, we used the network to identify relevant biological processes and molecular mechanisms that may be missing in our current pathway collections. All code and analysis are available here: https://gitlab.lcsb.uni.lu/computational-modelling-and-simulation/pathway-analysis-and-extension.

#### 5.1.4 Single-cell transcriptomic data analysis in lung epithelial cells of COVID-19 patients

In this section, we provided scRNA-seq gene expression analysis results to explore DEGs in specific lung epithelial cell populations in the COVID-19 patient group (moderate, severe, and critical cases), comparing with corresponding epithelial cell types isolated from the lungs of healthy subjects. The gene expression data was derived from scRNA-seq analysis of bronchoalveolar lavages from nine COVID-19 patients (three moderate, one severe, and five critical) (GSE145826) from (24). scRNA-seq data of epithelial cells (DAPI-, CD45-, CD31-, CD326+) isolated from control lung explant tissue of nine healthy subjects was used as a healthy control specific for lung epithelial cell types (25). All filtered samples were merged in one filtered gene-barcode matrix and analysed with the R package Seurat v.3 (84). The first 50 dimensions of canonical correlation analysis (CCA) and principal component analysis (PCA) were used in parameter settings. Moreover, the filtered gene-barcode matrix was first normalised using ‘LogNormalize’ method with default parameters. UMAP was performed on the top 50 PCs to visualise the cells, while clustering was performed on the PCA-reduced data for clustering analysis with Seurat v.3. The resolution was set to 0.5. A UMAP embedding represents the distribution of primary cell types in the scRNA-seq database (**Supplementary Figure S6**). The lung epithelial cell group (TPPP3, KRT18), directly infected by SARS-CoV-2, was analysed for every patient group. At first, the classification was provided, following these gene markers, as reported in (24): macrophages (CD68), neutrophils (FCGR3B), myeloid dendritic cells (mDCs; CD1C, CLEC9A), plasmacytoid dendritic cells (pDCs; LILRA4), natural killer (NK) cells (KLRD1), T cells (CD3D), B cells (MS4A1), plasma cells (IGHG4) and epithelial cells (TPPP3, KRT18). For the finest cell annotation of epithelial cell types, specific gene markers were used as reported in the Human Protein Atlas database (https://www.proteinatlas.org/), and markers of health epithelial cells reported by Deprez and colleagues (85) (10.1164/rccm.201911-2199OC) and extracted. In particular, ciliated cells (CFAP157, FAM92B; SARS-CoV-2-infected cells 15.5%), Secretory cells (BPIFB1, SCGB1A1, SCGB3A1; SARS-CoV-2-infected cells 6.4%), Suprabasal cells (KRT5, SERPINB4, KRT19, COVID19 cells 37.7%), Alveolar Type 1 cells (AGER, CAV1, EMP2, SARS-CoV-2-infected cells 6%), Basal cells (KRT5, KTR15, COVID19 cells 11.2%). Alveolar Type 2 cells were not included because of an unbalanced ratio of cell sample size between COVID-19 cases and healthy control (SARS-CoV-2-infected cells <2%; see **Supplementary Table S4** for a detailed summary of all cell types). The balanced sample size of cells allowed us to compare these two groups. Differential gene expression analysis between patients and specific cell control was performed for epithelial cell groups. A differential gene expression analysis for all clusters was performed using the FindMarkers function in Seurat v.3, imposing a statistical threshold of 0.05% FDR, average |logFC| > 1 and the difference between PCs>0.25 to increase confidence in the results. All code and analysis are available here: https://gitlab.lcsb.uni.lu/computational-modelling-and-simulation/single-cell-transcriptomic-data-analysis-in-epithelial-cell-types-of-covid-19-patient-groups-with-different-severity-profiles.

#### 5.1.5 Integrative pathway modelling using C19DMap diagrams and RNAseq data from COVID-19 patients

The HiPathia algorithm allows modelling the behaviour of signalling pathways, described as directed graphs that connect receptor proteins to effector proteins through a chain of activations and inhibitions exerted by intermediate proteins. HiPathia treats the pathways as if they were composed of elementary circuits, each circuit defined as the sub-pathway, or chain of proteins, connecting receptors to effectors. HiPathia uses expression values of genes as proxies of the activation levels of the corresponding proteins in the circuit (86). To estimate the activity of a given circuit, a signal value of 1 is transmitted through the ⁠nodes and modulated by the activity values of the intervening proteins until it reaches the final effector protein, which is annotated with the functions it triggers in the cell (27). These circuit activation values can be assessed between conditions to obtain differential signalling and functional activity profiles. The first version of the C19DMap has been implemented in the CoV-HiPathia version (87). In addition, extracted SIF files from SBML qual files using CaSQ (33) can be imported to HiPathia containing the Activity Flow (AF) structure of the Process Description (PD) diagrams, enabling new disease maps to be modelled as they are built, thus permitting their exploration and analysis. In order to test the methodology, a public RNAseq dataset of nasopharyngeal swabs from 430 individuals with SARS-CoV-2 and 54 negative controls (26) (GSE152075) was used. First, the RNA-seq gene expression data were normalised with the Trimmed mean of M values (TMM) normalisation method using the edgeR R package (88)⁠. Then, within the CoV-Hipathia web tool (87)⁠, the HiPathia algorithm requires the expression data to be rescaled between 0 and 1 to calculate the signal. Finally, quantile normalisation was done using the preprocessCore R package (86). The normalised gene expression values were used to calculate the level of activation of the sub-pathways, and then a case/control contrast with a Wilcoxon test was used to assess differences in signalling activity between the two conditions: SARS-CoV-2-infected and normal control nasopharyngeal tissue (FDR adjusted p-value < 0.05). Data and code available: https://gitlab.lcsb.uni.lu/computational-modelling-and-simulation/Hipathia_IFN1_Renin-Angiotensin_analysis.

### 5.2 Dynamical modelling at the molecular, cellular, and multicellular levels

#### 5.2.1 Dynamical modelling of type 1 IFN responses in SARS-CoV-2 infection

##### 5.2.1.1 Type 1 IFN model development and computational validation

We used the type 1 IFN molecular map as a scaffold and auto-generated the dynamic model using the CaSQ tool. We evaluated the model’s behaviour using seven biological scenarios from the scientific literature.

##### 5.2.1.2 Global sensitivity analysis

We simulated the model in Cell Collective (37) using varying activity levels of each input. We determined the input-output association using activity levels of 1000 randomly-generated simulations as previously used by our group (89). We performed probabilistic global sensitivity analysis based on the partial correlation coefficient (PCC) using the “sensitivity” package (https://cran.r-project.org/web/packages/sensitivity/sensitivity.pdf) in R (R Core Team, 2016) on data obtained from Cell Collective. It shows the impact of change in the input variable (independent variable) on the output variable (dependent variable) while considering and removing the linear effect of other input variables on the output variable (90). The script used in this analysis is available in our shared GitLab repository (https://git-r3lab.uni.lu/computational-modelling-and-simulation/analysis/-/blob/master/IFN1_modelling/Global_Sensitivity_analysis_of_IFN_model.R).

##### 5.2.1.3 Sensitivity analysis against overexpression and knockouts

The sensitivity of biomolecules was calculated against knockout and overexpression perturbations. The sensitivity values were quantified in macro values for each biomolecule. The bitwise distances were calculated for each biomolecule in the same macro class. The highest sensitivity values were then simulated in Cell Collective. The methodology of the algorithm used to calculate the sensitivities against knockout and over-expression perturbations is described in FairdomHub (https://fairdomhub.org/data_files/4090), and the used script that generates the result is available in our shared GitLab repository (https://git-r3lab.uni.lu/computational-modelling-and-simulation/analysis/-/blob/master/IFN1_modelling/IFN1_sensitivity_against_mutations.R).

##### 5.2.1.4 Input propagation for calculating stable states

The IFN model has 55 input components. These input components maintain their activity level as they have no upstream regulators, and their initial configuration plays a vital role in the potential outcome. We consider that all inputs representing viral components share a common state to eliminate unrealistic input configurations. To encode this constraint, we introduce an additional input node controlling this group of components. We applied the same approach to the immune response and IFN secretion inputs. In the resulting model, only six inputs remain, these three meta-inputs and three components representing drugs (GRL0617, Azithromycin, and MNS). Using this modified model, we identified 128 stable states. The absence of other stable patterns suggests that this model does not generate stable oscillations. We selected four output components to assess the obtained phenotypes (viral replication, antiviral response, inflammation, and secretion of IFNA1). The projection of the 128 stable states on these four outputs gave six distinct signatures among the 16 possibilities. All signatures lacked IFN secretion and exhibited either viral replication or antiviral response (or both). We then studied in more detail a set of 8 input conditions that cover different biological scenarios of the type 1 IFN pathway with or without the infection and in the presence or absence of drugs (**Supplementary Table S2**). In these conditions, the propagation of the input values was sufficient to control most components of the model, particularly all selected output components. Studies in patients with COVID-19 with various degrees of severity showed hampered IFN-I responses in patients with severe or critical COVID-19. These patients had low levels of IFN-I and ISGs and increased production of TNF-, IL-6-, and NF-κB-mediated inflammation. All code and analysis are available here: https://gitlab.lcsb.uni.lu/computational-modelling-and-simulation/analysis.

#### 5.2.2 Integration of the Type I IFN, the ACE-ACE2 axis, and the NLRP3 inflammasome curated pathways into a macrophage-specific Boolean model

Three diagrams in the C19DMap repository were selected: the Type I IFN, the ACE-ACE2 axis, and the NLRP3 inflammasome. These diagrams were converted into SMBL qual formats using the CaSQ tool (33) and then processed in GINsim (61). Once processed, the pathway modules were integrated into a COVID-19-specific macrophage model. Phenotypic nodes were added to link the biomarkers with a biological process easily using an associated GO term name. Next, the functionality and behaviour of the COVID-19 macrophage model were evaluated in a stable state analysis (attractors) performed with the following stimulatory conditions: inflammatory microenvironment, virus infection, and both. https://gitlab.lcsb.uni.lu/computational-modelling-and-simulation/macrophage-model.

#### 5.2.3 Multiscale and multicellular simulation

We incorporated two Boolean models into a multiscale simulator that consists of the infection of a patch of lung epithelium by SARS-CoV-2 and the immune cells that are recruited (45): macrophages, neutrophils, dendritic cells, CD4- and CD8-T-cells. We expanded this simulator with our tool, PhysiBoSS (91), which incorporates MaBoSS (92), a tool that stochastically simulates Boolean models, into PhysiCell (93), a tool that uses agent-based modelling to simulate cells and their surrounding environment, and their interplay. Two Boolean models were used: first, the epithelial apoptosis model was converted from the map to the model using CaSQ (33) and the C19DMap project (https://fairdomhub.org/models/712) (82). We modified the apoptosis model to capture mechanisms such as BAX activating the apoptosome complex and included output nodes as readouts. We also connected inputs and outputs to different variables in the population model, such as the *Virus_inside* node, which depends on the number of virions inside a cell, or the *Tcell_attached* node, which depends on the attachment of a T-cell to the epithelial cell (**Figure 7C**). Second, we included the macrophage-specific Boolean model developed for this work. As with the apoptosis model, we connected the models’ inputs and outputs to relevant variables from the agents. For instance, we activated the *Apoptotic_cell* node upon encountering an apoptotic epithelial cell, activated the *SARS_CoV_2* node upon encountering a virion, or activated the interferon Boolean nodes when the interferon roaming in the environment was above the detection threshold. Likewise, when *Neutrophil_recruitment*, *CD4_Tcell_activation* or *CD8_Tcell_activation* nodes are ON, pro-inflammatory cytokines are released. We found perturbations in the Boolean model that enhanced the recruitment of immune cells and the commitment to apoptosis using our pipeline of tools (46) that uses MaBoSS to simulate stochastic trajectories. All code and analysis are available here: https://gitlab.lcsb.uni.lu/computational-modelling-and-simulation/pb4covid19.

### 5.3 Pharmacogenomic analysis

We obtained the list of proteins in the C19DMap as well as lists of proteins targeted by drugs and chemicals from annotations from the AILANI COVID-19 research assistant (https://ailani.ai) based on an NLP pipeline (54), INDRA (Integrated Network and Dynamical Reasoning Assembler) (4), and from the Clinical Trials DB. We used information from the cross-references from DrugBank (78) to map ChEBI and PubChem identifiers to DrugBank identifiers. We further enriched the list of drug/chemical targets using the information from DrugBank (accessed June 2022). A list of 16 drugs used to treat COVID-19 was obtained from (94), and their targets were obtained from DrugBank. After merging the lists, a final dataset of 1,476 drugs and chemicals (identified by DrugBank IDs) and 1,120 drug targets (identified by NCBI Gene ID) was obtained. Information on pharmacogenomic variants for the drug targets was retrieved from PharmGKB (95) (accessed on Feb 14, 2021). For each gene that encodes a drug target, the list of variants with pharmacogenomic annotations that are significant and are annotated to a dbSNP identifier was retrieved. We used the cross-references from PharmGKB to map the PharmGKB drug accessions to DrugBank identifiers. Data on the allelic frequency of the pharmacogenomic variants were retrieved from The Genome Aggregation Database (gnomAD) (96) (version 2.1.1). gnomAD is a resource developed by an international coalition of investigators to aggregate and harmonise exome and genome sequencing data from various large-scale sequencing projects and make summary data available for the broader scientific community. To aggregate the data on the pharmacogenomic impact and allelic frequency of the variants, we computed a modified version of the Cumulative Allele Probability (CAP) and the “Drug Risk Probability” (DRP) score (50). The CAP score considers the number of pharmacogenomic variants and their frequency in the population for a specific gene. The DRP score combines the CAP scores for all drug target genes for a specific drug. The code to compute the CAP and DRP scores is available at https://github.com/jpinero/pharmacogenomics_covid19_minerva_map/.

### 5.4 AI-assisted map updating and expanding

All INDRA code and analyses are provided here: https://github.com/indralab/covid-19/tree/master/covid_19/disease_maps.

AILANI results have been integrated into the resources files: https://git-r3lab.uni.lu/covid/models/-/tree/master/Resources/Expand%20the%20diagrams.

### 5.5 Topological analysis

We calculated values for 17 network centrality measures for each available pathway as implemented in Vanted’s Centilib extension (97). Taking into account the results of correlation analysis and the requirements of centrality calculation on the network structure, such as connectivity, we restricted the 17 measures to a base set of 10 measures (Eccentricity, Degree, Eigenvector, HITSAuths, Current Flow Betweenness, Radiality, Stress, Shortest Path Betweenness, Centroid Rank, Closeness) (98). We calculated the values for each network node (excluding reactions) for these measures and provided rankings of nodes for each measure per network. Additionally, we computed aggregated rankings using the residual sum of squares for each node per network and on the aggregated network. The results from our centrality calculations can also be explored and put in context using the software LMME-DM (https://github.com/LSI-UniKonstanz/lmme-dm) developed as part of the C19DMap project. It follows an overview and detail approach, showing an overview graph containing one node per pathway and a detailed pathway view, including the detailed crosstalks. The centrality values can now be mapped on the nodes’ size and colour (see **Figure 9**). All code and analysis are available here: https://gitlab.lcsb.uni.lu/computational-modelling-and-simulation/graphical-exploration-and-topological-analysis.

### 5.6 C19DM-Neo4j database

The input maps were gathered from the COVID-19 Disease Map curation repository (https://git-r3lab.uni.lu/covid/models, October 2020, commit a705765a). All stable maps stored in the CellDesigner format were considered for 21 maps. The maps were first converted from the CellDesigner format to SBGN-ML using the CD2SBGML tool. The conversion resulted in 19 maps (maps “ETC_stable.xml” and “E_protein_stable.xml” could not be converted by CD2SBGNML). These maps were then stored in the Neo4j database using StonPy (99). All code and analysis are available here: https://gitlab.lcsb.uni.lu/computational-modelling-and-simulation/c19dm-neo4j-db.

### 5.7 Orthoinference process for converting from SARS-CoV-1 to SARS-CoV-2 diagrams

The standard orthoinference process is used in the Reactome database to infer reactions electronically in fifteen evolutionarily divergent eukaryotic species for which high-quality whole-genome sequence data are available. Eligible reactions are checked to determine whether each involved protein has at least one homologous protein in the reaction’s input, output, and (if present) catalyst in the organism undergoing inference. If a human reaction involves a complex, at least 75% of the accessioned protein components of the human complex must have homologous proteins in the model organism. The first (V74) draft of this SARS-CoV-2 pathway consists of 101 reactions involving 489 molecular entities (279 proteins, 12 RNAs, and 198 others) and is supported by citations from 227 publications. Reactome developed a computational triaging strategy to review and identify publications appropriate for manual curation (66,100 SARS-CoV-2 articles on PUBMED, tallied on 30/October/2020).

## Data availability statement

The original contributions presented in the study are included in the article/**Supplementary Materials** and in the gitlab repository https://gitlab.lcsb.uni.lu/computational-modelling-andsimulation/, further inquiries can be directed to the corresponding author/s.

## Ethics statement

Ethical approval was not required for the studies involving humans because this is a meta-analysis of publicly available data. The studies were conducted in accordance with the local legislation and institutional requirements. We only analyzed publicly available datasets. Written informed consent to participate in this study was not required from the participants or the participants’ legal guardians/next of kin in accordance with the national legislation and the institutional requirements.

## Author contributions

AN: Conceptualization, Data curation, Formal analysis, Investigation, Methodology, Project administration, Resources, Software, Supervision, Visualization, Funding acquisition, Writing – original draft, Writing – review & editing. MO: Conceptualization, Data curation, Funding acquisition, Investigation, Methodology, Project administration, Resources, Software, Supervision, Visualization, Writing – original draft, Writing – review & editing. AM: Data curation, Methodology, Software, Writing – review & editing. IK: Data curation, Writing – review & editing. MK: Data curation, Formal analysis, Investigation, Methodology, Visualization, Writing – review & editing. MG: Data curation, Investigation, Methodology, Resources, Visualization, Writing – review & editing. AF: Data curation, Formal analysis, Investigation, Methodology, Software, Writing – review & editing. MA: Data curation, Investigation, Writing – review & editing. AH: Data curation, Formal analysis, Investigation, Methodology, Writing – review & editing. MAi: Data curation, Formal analysis, Investigation, Methodology, Software, Visualization, Writing – review & editing. KK: Data curation, Formal analysis, Investigation, Methodology, Software, Visualization, Writing – review & editing. TC: Data curation, Formal analysis, Investigation, Methodology, Software, Visualization, Writing – review & editing. FB: Data curation, Investigation, Writing – review & editing. TY: Data curation, Formal analysis, Investigation, Methodology, Writing – review & editing. YH: Data curation, Formal analysis, Investigation, Methodology, Writing – review & editing. NH: Data curation, Formal analysis, Investigation, Methodology, Writing – review & editing. FH: Data curation, Formal analysis, Investigation, Methodology, Visualization, Writing – review & editing. NP: Data curation, Formal analysis, Investigation, Methodology, Visualization, Writing – review & editing. FE: Data curation, Formal analysis, Investigation, Methodology, Visualization, Writing – review & editing. EW: Data curation, Formal analysis, Investigation, Methodology, Visualization, Writing – review & editing. AV: Data curation, Formal analysis, Investigation, Methodology, Software, Visualization, Writing – review & editing. AD: Data curation, Formal analysis, Investigation, Methodology, Software, Visualization, Writing – review & editing. FM: Data curation, Formal analysis, Investigation, Methodology, Visualization, Writing – review & editing. ME: Data curation, Formal analysis, Investigation, Methodology, Software, Visualization, Writing – review & editing. MP-C: Data curation, Formal analysis, Investigation, Methodology, Software, Visualization, Writing – review & editing. KR: Data curation, Formal analysis, Investigation, Methodology, Software, Visualization, Writing – review & editing. SS: Data curation, Formal analysis, Investigation, Methodology, Software, Writing – review & editing. SA: Data curation, Formal analysis, Investigation, Methodology, Visualization, Writing – review & editing. BP: Data curation, Formal analysis, Investigation, Methodology, Visualization, Writing – review & editing. ANa: Data curation, Formal analysis, Investigation, Methodology, Software, Visualization, Writing – review & editing. TH: Formal analysis, Investigation, Methodology, Software, Visualization, Writing – review & editing. VS: Data curation, Formal analysis, Methodology, Writing – review & editing. MF: Data curation, Formal analysis, Methodology, Visualization, Writing – review & editing. VB: Data curation, Formal analysis, Methodology, Visualization, Writing – review & editing. ET: Data curation, Formal analysis, Methodology, Visualization, Writing – review & editing. AMo: Data curation, Formal analysis, Investigation, Methodology, Software, Visualization, Writing – review & editing. VN: Data curation, Formal analysis, Investigation, Methodology, Software, Visualization, Writing – review & editing. MP: Data curation, Formal analysis, Investigation, Methodology, Software, Visualization, Writing – review & editing. DM: Data curation, Formal analysis, Investigation, Methodology, Software, Writing – review & editing. AB: Data curation, Formal analysis, Investigation, Methodology, Software, Writing – review & editing. BG: Data curation, Investigation, Methodology, Software, Writing – review & editing, Formal analysis, Resources. JB: Data curation, Formal analysis, Investigation, Methodology, Resources, Software, Writing – review & editing. AL: Data curation, Investigation, Methodology, Writing – review & editing. JP: Data curation, Formal analysis, Investigation, Methodology, Visualization, Writing – review & editing. LF: Data curation, Formal analysis, Investigation, Methodology, Visualization, Writing – review & editing. IB: Data curation, Investigation, Methodology, Visualization, Writing – review & editing. AR: Data curation, Investigation, Methodology, Visualization, Writing – review & editing. YJ: Data curation, Investigation, Methodology, Visualization, Writing – review & editing. RO: Data curation, Investigation, Writing – review & editing. RP: Data curation, Investigation, Writing – review & editing. LP: Data curation, Investigation, Writing – review & editing. LM: Data curation, Investigation, Methodology, Visualization, Writing – review & editing. DR: Data curation, Writing – review & editing. MO-M: Data curation, Writing – review & editing, Investigation, Methodology, Visualization. CL: Data curation, Methodology, Writing – review & editing. BD: Data curation, Writing – review & editing, Investigation. JR: Data curation, Writing – review & editing, Methodology. BJ: Data curation, Methodology, Writing – review & editing, Investigation, Resources. VSa: Investigation, Methodology, Resources, Writing – review & editing. GW: Methodology, Writing – review & editing, Data curation, Software, Visualization. MGo: Writing – review & editing, Resources. PG: Resources, Writing – review & editing, Methodology, Software. LC: Methodology, Software, Writing – review & editing, Data curation, Formal analysis, Investigation. JBe: Data curation, Investigation, Writing – review & editing, Resources. CE: Investigation, Resources, Writing – review & editing, Methodology. PD: Investigation, Methodology, Resources, Writing – review & editing, Data curation. FS: Methodology, Resources, Writing – review & editing, Formal analysis, Software. JS-R: Methodology, Resources, Software, Writing – review & editing. JD: Methodology, Resources, Software, Writing – review & editing. MKui: Methodology, Resources, Writing – review & editing, Investigation. AlfV: Writing – review & editing, Resources, Funding acquisition. OW: Funding acquisition, Resources, Writing – review & editing. HK: Funding acquisition, Resources, Writing – review & editing. EB: Funding acquisition, Resources, Writing – review & editing. CA: Funding acquisition, Resources, Writing – review & editing. RB: Funding acquisition, Resources, Writing – review & editing. RS: Funding acquisition, Resources, Writing – review & editing.

## Footnotes

1
https://covid19.who.int/.

2
https://www.who.int/news/item/05-05-2023-statement-on-the-fifteenth-meeting-of-the-international-health-regulations-(2005)-emergency-committee-regarding-the-coronavirus-disease-(covid-19)-pandemic.

3
https://clinicaltrials.gov/ct2/show/NCT04803227 ?term=emricasan&draw=4&rank=4.
