# Supplementary material for: Drug-target identification in COVID-19 disease mechanisms using computational systems biology approaches
Source: Front Immunol. 2024 Feb 13;14:1282859. doi: 10.3389/fimmu.2023.1282859 (PMC10897000; doi:10.3389/fimmu.2023.1282859)
Supplement: Supplementary file 2 [file DataSheet_2.pdf]

Supplementary Material for

Title: Drug-Target identification in COVID-19 disease mechanisms using computational systems biology approaches

DOI: 10.3389/fimmu.2023.1282859

Anna Niarakis *et al*.

*Corresponding author. Email: anna.niarakis@inria.fr

This PDF file includes:

Figs. S1 to S17
Tables S1 to S17 with the exception of Tables S8 and S9 that are provided in a separate excel file.

Fig. S1.

A) Carnival network of mechanistic hypotheses connecting the top 10 deregulated kinases with the top 30 deregulated TFs. Kinases are represented as hexagon-shaped nodes, TFs as rounded rectangles, and intermediary signaling proteins as elliptical nodes. Up-regulated nodes are indicated in red while down-regulated nodes are colored in blue. Activatory reactions are indicated with arrow-shaped edges whereas inhibitory ones are represented by T-shaped edges. Bold outlines denote proteins already present in the COVID-19 Disease Map. Blue outlines denote PAMPs and Interferon-1 pathway members, red outlines are ER stress pathway members and purple outlines indicate TGFbeta pathway members of the pathways manually curated by the COVID-19 Disease Maps community. B) Highlighted C19DMap diagrams containing deregulated kinases and TFs are shown in dark pink. Users can click on the highlighted boxes and enter the diagram.

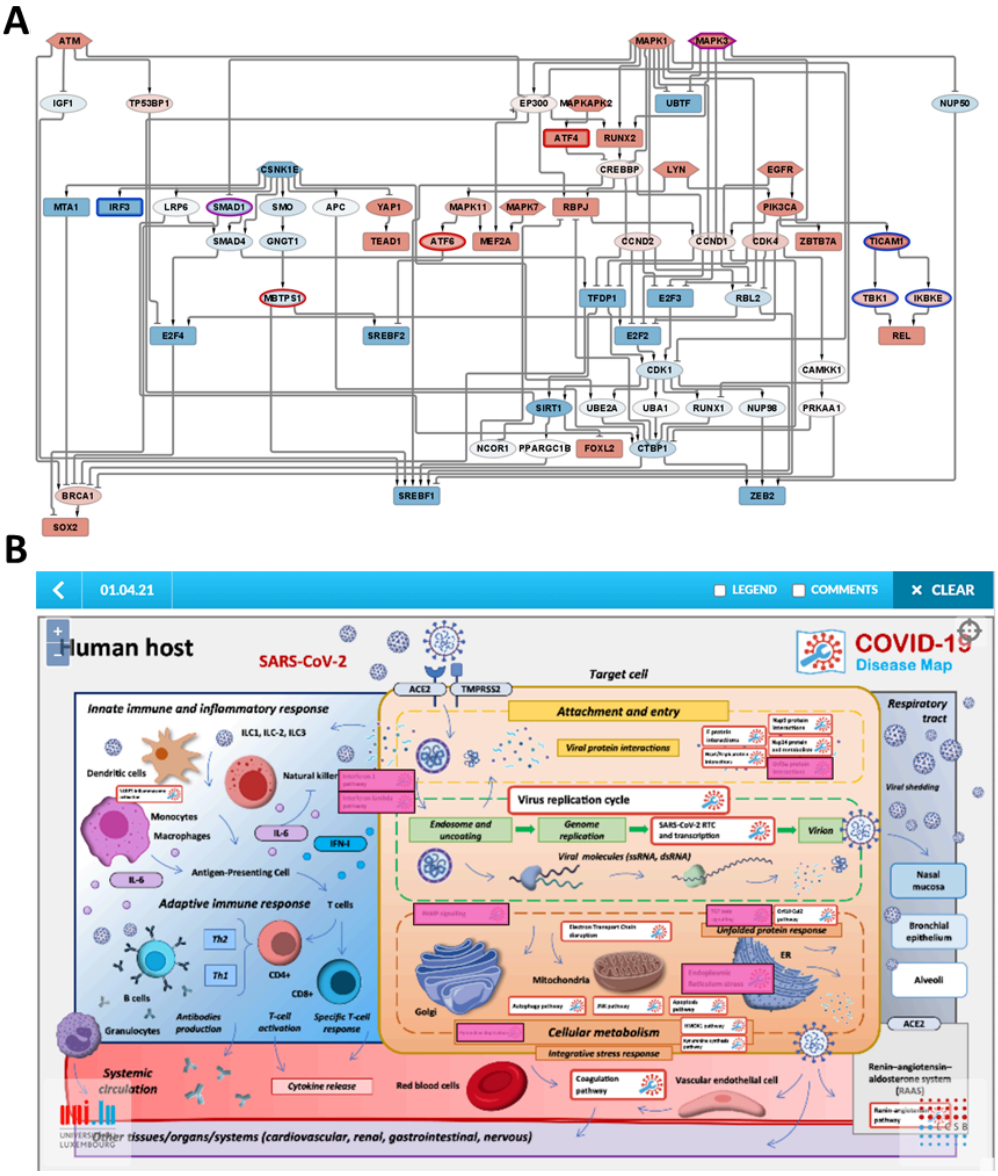

Fig. S2.

Overlap of (a) DEGs and (b) TFs between SARS-CoV-2-infected NHBE and A549 cells by LAMP and GO Enrichment analysis.

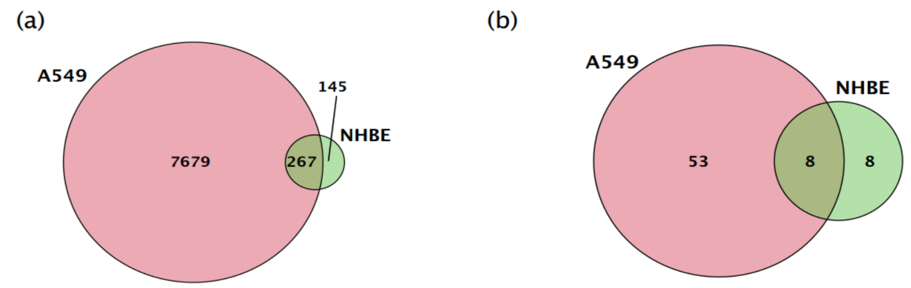

Fig. S3.

(A) Venn diagram of the combined pathway gene sets from COVID-19 Disease Map, WikiPathways, and Reactome with 1,840 human pathways containing 12,037 unique genes. (B) Over-representation analysis (criteria: absolute fold change > 1.5 and p-value < 0.05) revealed 11 pathways altered in both cell lines. WP = WikiPathways

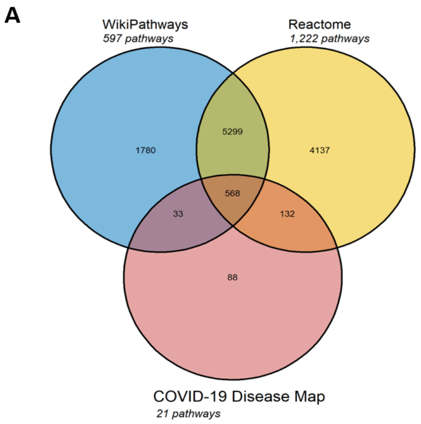

B

No	Altered pathways in NHBE and A549 cell lines after SARS-CoV-2 infection
1	WP_Photodynamic therapy-induced NF-kB survival signaling
2	REACTOME_INTERLEUKIN_10_SIGNALING
3	WP_Selenium micronutrient network
4	REACTOME_INTERLEUKIN_4_AND_INTERLEUKIN_13_SIGNALING
5	WP_Immune response to tuberculosis
6	WP_Folate metabolism
7	WP_IL1 and megakaryocytes in obesity
8	WP_Antiviral and anti-inflammatory effects of Nrf2 on SARS-CoV-2 pathway
9	WP_Nuclear receptors meta-pathway
10	REACTOME_EXTRACELLULAR_MATRIX_ORGANIZATION
11	WP_Glucocorticoid receptor pathway

Fig. S4.

C19DMap (DM), WikiPathways (WP) and Reactome pathways significantly enriched for DEGs in SARS-CoV-2-infected NHBE cells.

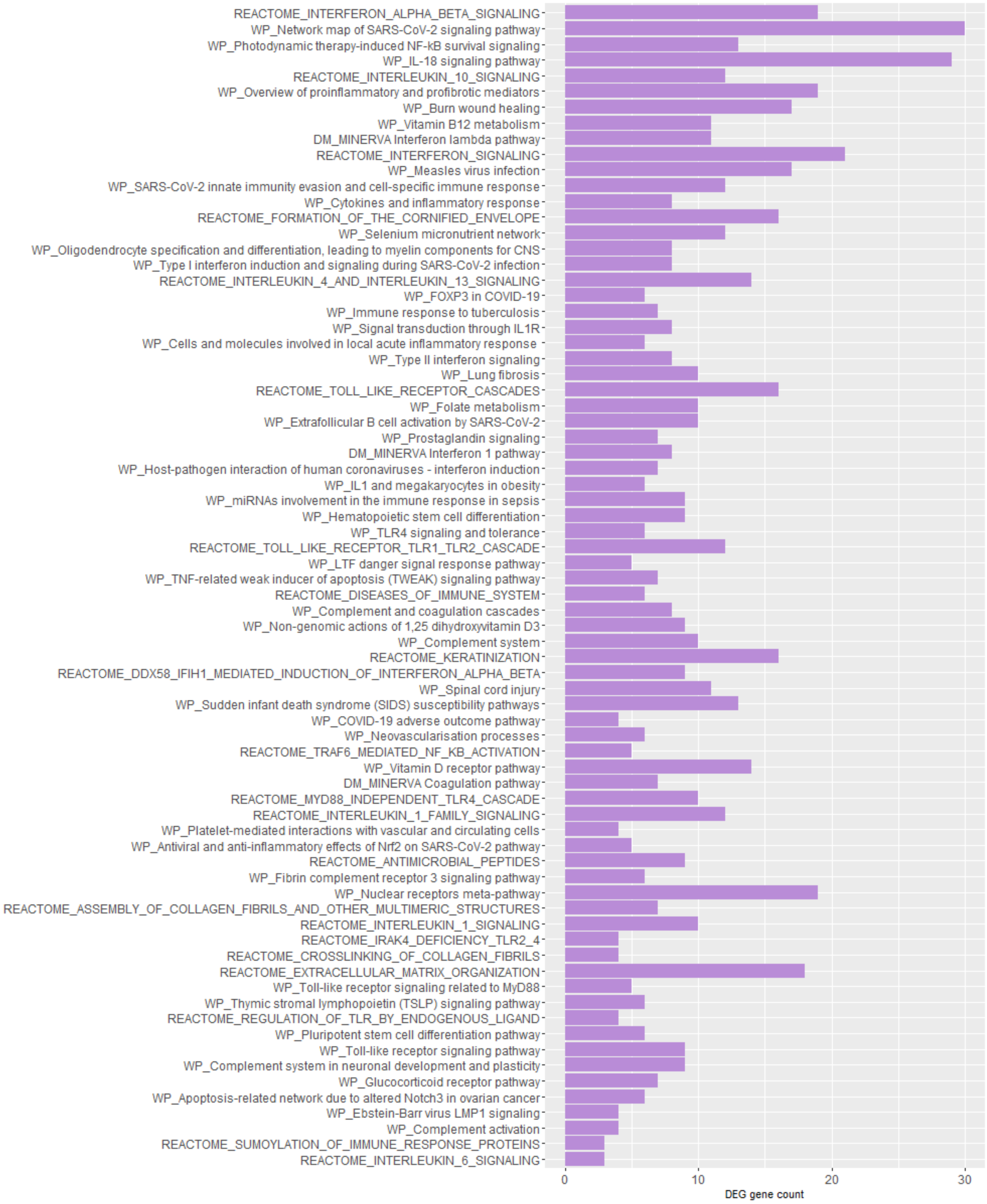

Fig. S5.

C19DMap (DM), WikiPathways (WP) and Reactome pathways significantly enriched for DEGs in SARS-CoV-2-infected A549 cells.

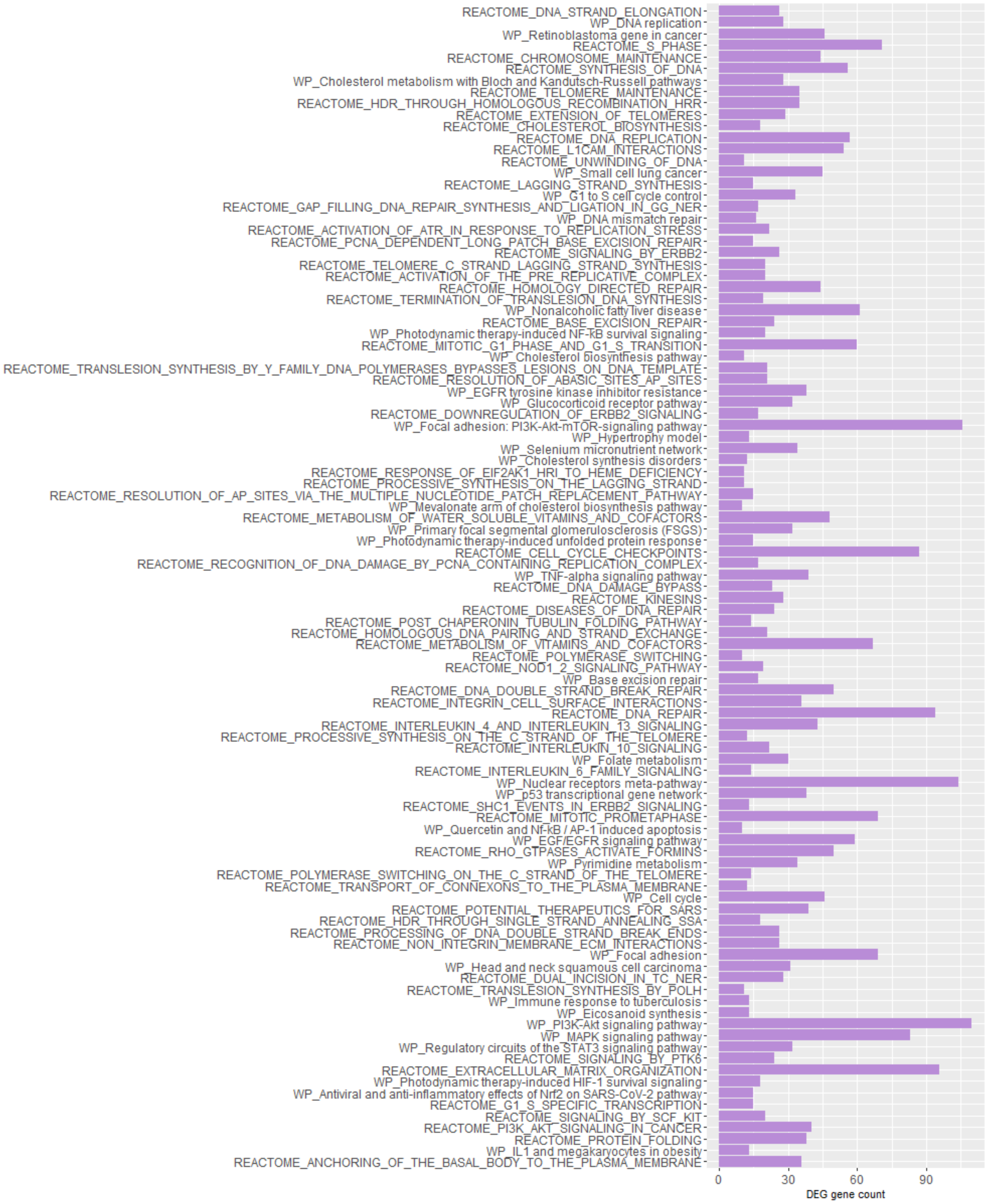

Fig. S6.

A) UMAP of both whole data sets (GSE145926 and GSE160664), where all cell types were highlighted and reported. B) Overlap of overexpressed (positive) DEGs among all detected lung epithelial cell types of COVID-19 patients relative to healthy controls.

A)

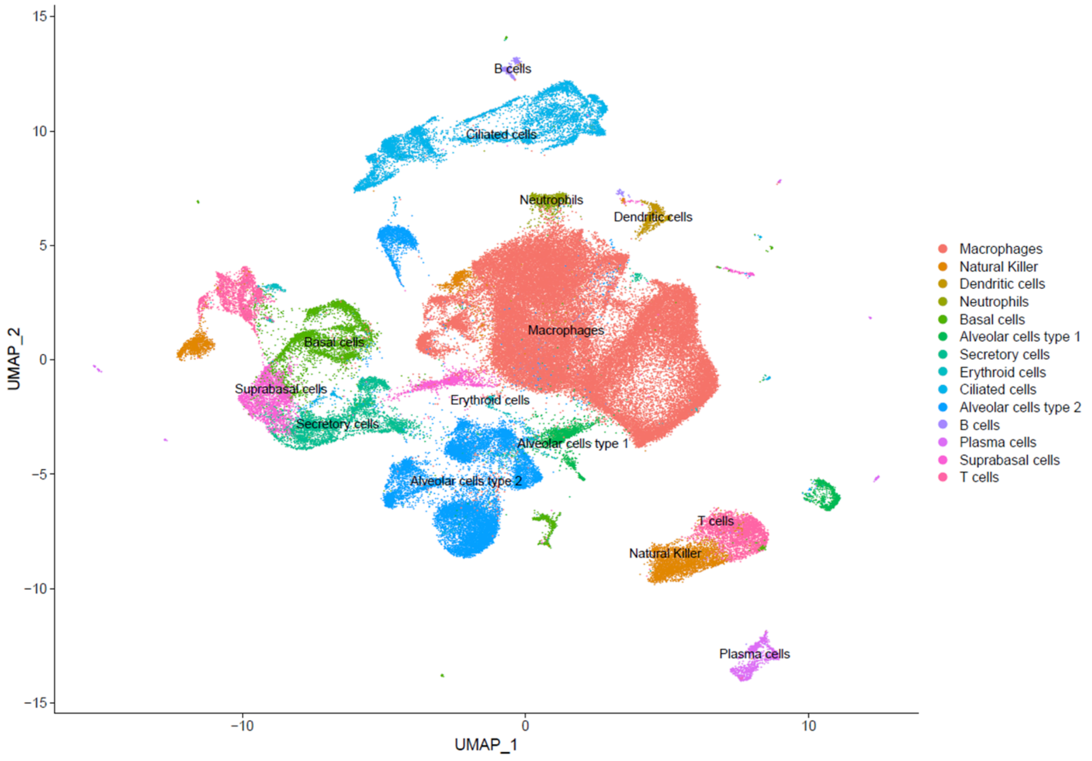

B)

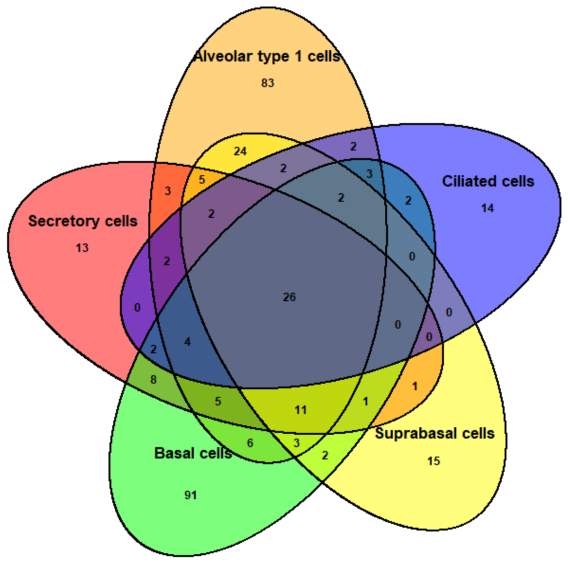

Fig. S7.

The Boolean network obtained after the processing of the CellDesigner XML file of the Type I IFN of the C19DMap repository with CaSQ 9.0.2. After post-processing, the final version of the model contains 121 nodes and 190 edges. Colour code: Red: Viral proteins, Turquoise: Drugs, Pink: host cell inputs, Purple: Host cell proteins, Light blue: Phenotypes of interest

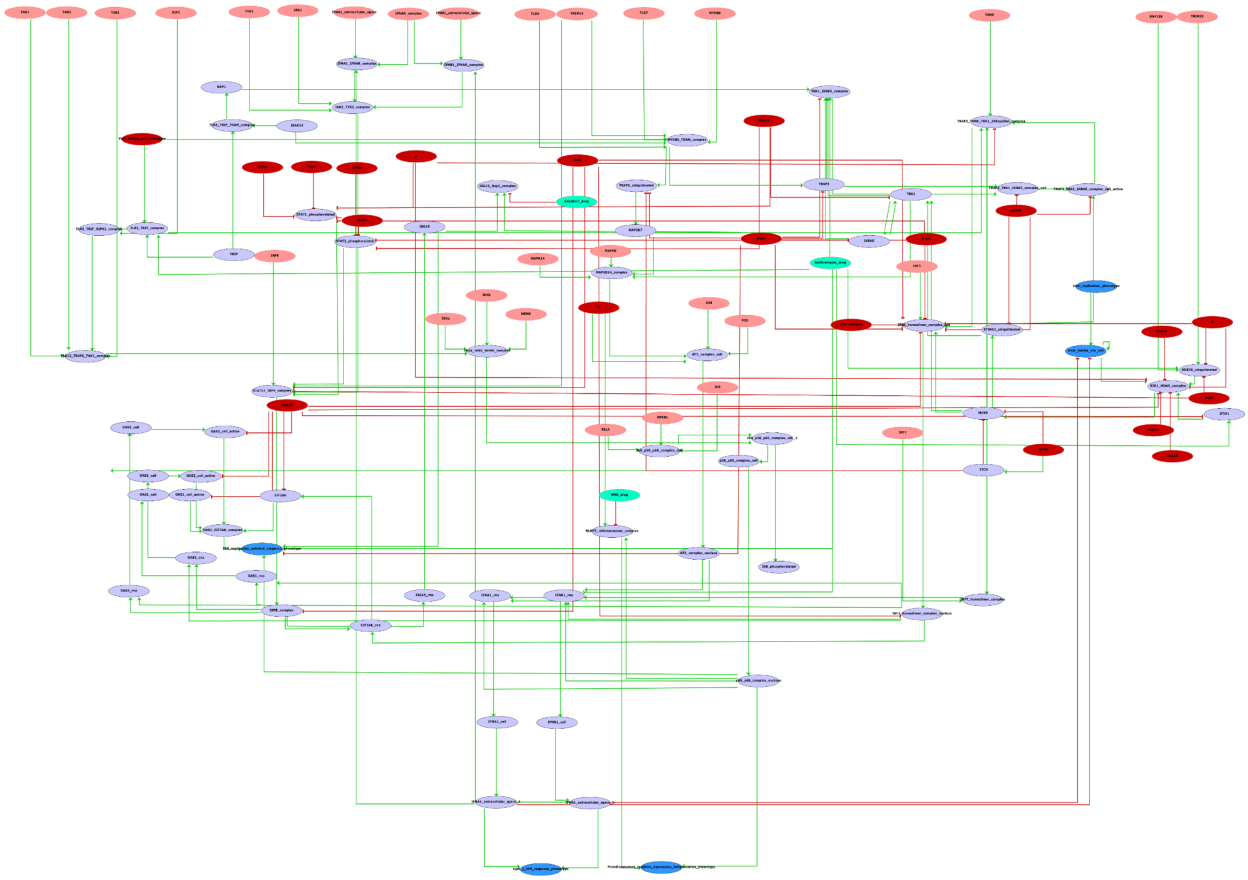

Fig. S8.

Example of the in-silico simulation in Cell Collective. The orange line presents the inflammation activity level. The left graph presents the increase of inflammation phenotype when viral components are activated after the time step 20. The right graph presents the decrease of inflammation phenotype in the presence of viral components upon addition of the GRL0617, Azithromycin, and MNS drugs to the system (at time step 20).

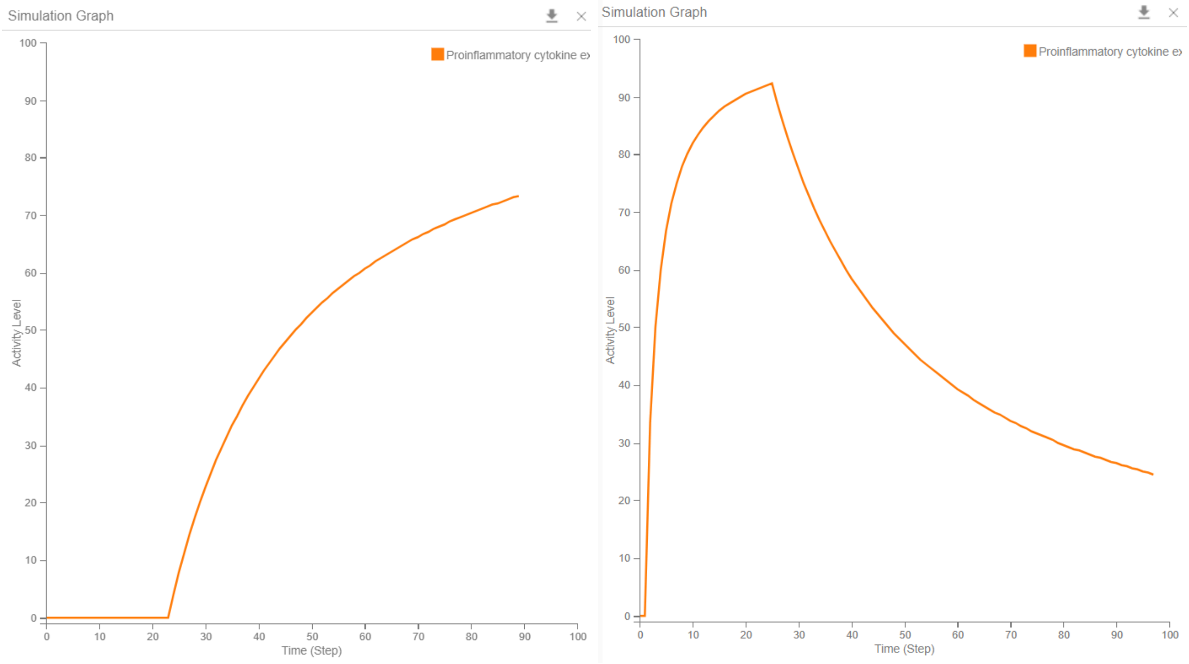

Fig. S9.

Sensitivity analysis showing the association between inputs (external components) and outputs (phenotypes). A) All the inputs, except drugs, were active. Inflammation phenotype has the highest positive association with protein E. The 'ISG expression antiviral response phenotype' has the highest association (negative) with Nsp3. B) All the inputs were active. The 'Inflammation phenotype' has the highest positive association with viral protein E and negative association with the MNS drug. An increase in viral proteins leads to higher inflammation. In contrast, the increase in MNS reduces inflammation. PCC is a partial correlation coefficient measuring the strength of association between external components and output variables.

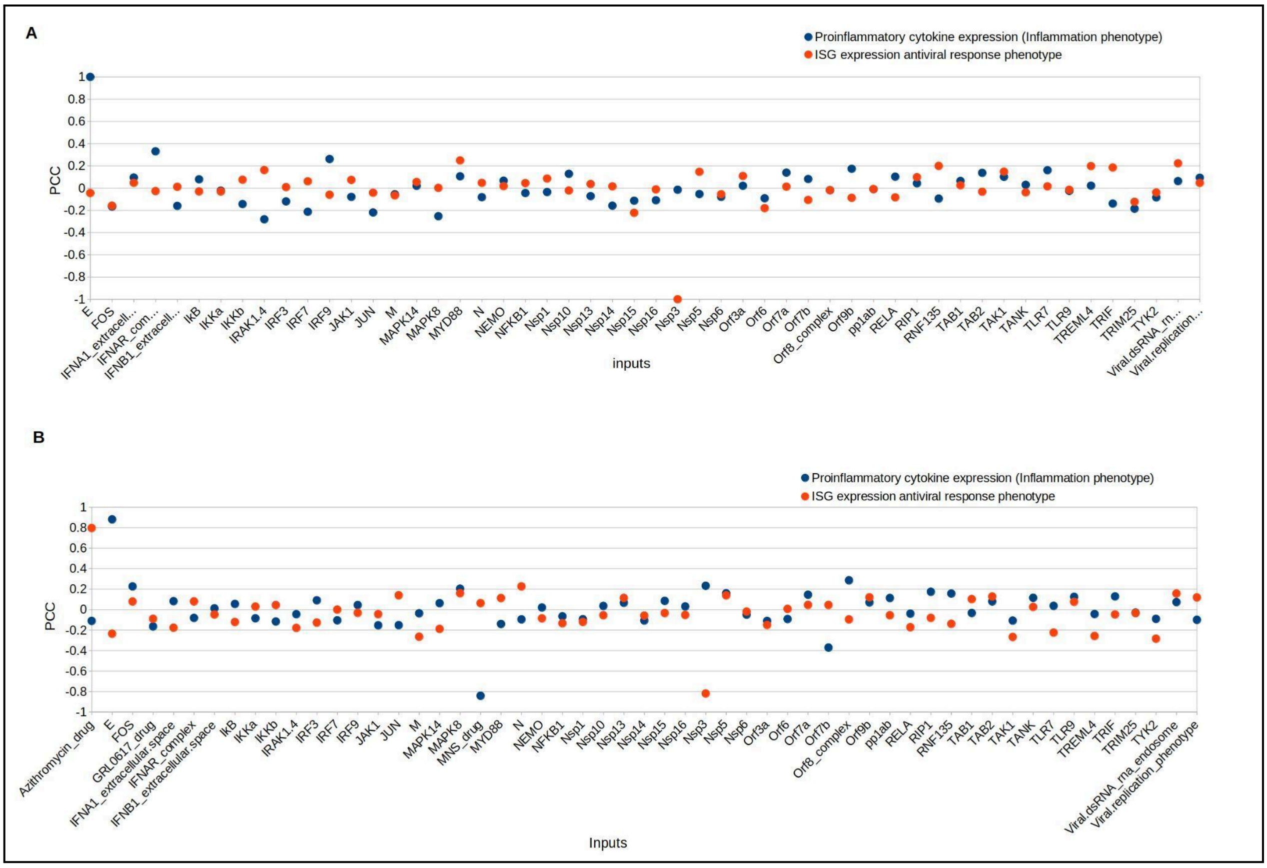

Fig. S10.

The result of input propagation can be visualized in the heatmap where columns represent components of the system and rows represent the 8 selected input conditions. A white cell denotes that the corresponding component is fixed at value 0 in this input condition. Likewise, a blue cell denotes that it is fixed at 1. Red cells denote components which are not fixed by input propagation.

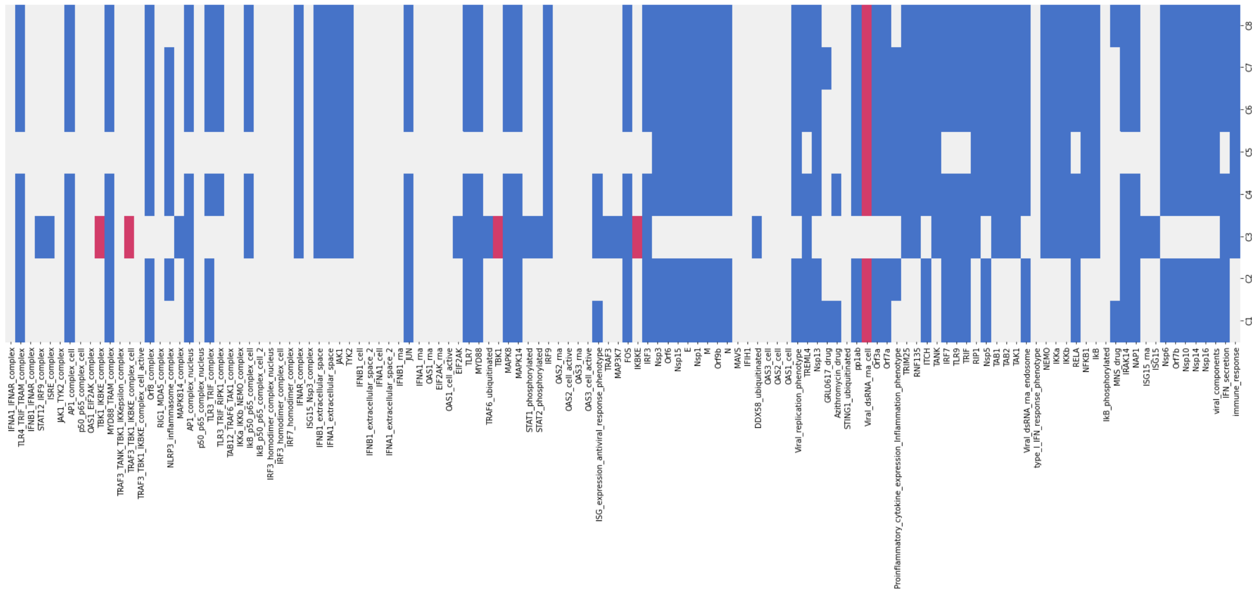

Fig. S11.

Close-up comparison of the effect of p38 knockout (KO) on the phenotype scores of the macrophages model personalized for patient C141. Phenotype scores of the recruitment of immune cells were gathered for the wild-type C141 (red) and the p38 knockout C141 MaBoSS simulation (blue).

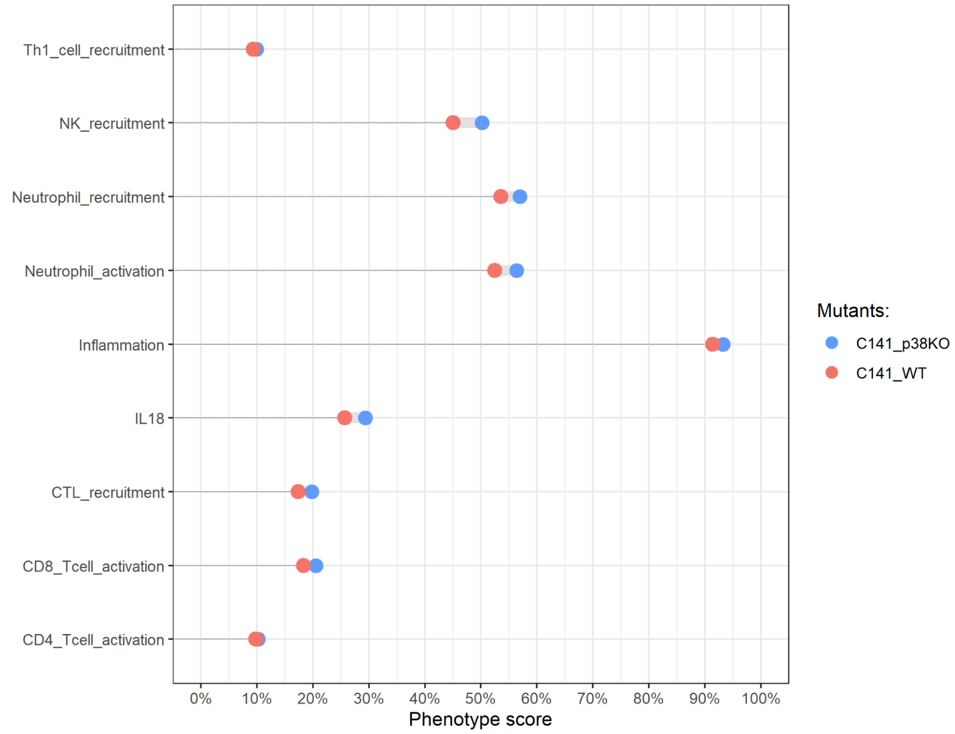

Fig. S12.

High-throughput study of all the KO mutants of the Boolean models considered in the multiscale simulations. The resulting scores for the relevant phenotypes apoptosis of respiratory epithelial cells (A) and the recruitment of immune cells by macrophages (B) were compared to the wild-type values (red bars). The green bar represents the score of FADD knockout, and the blue bar represents the score of p38 knockout.

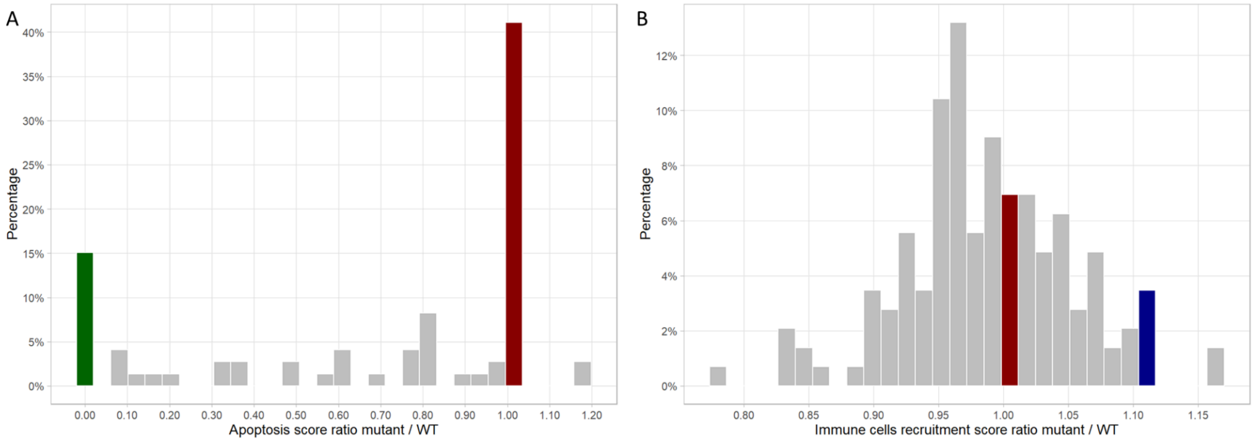

Fig. S13.

Simulation of wild type and mutants using PhysiBoSS. Our framework can simulate wild-type respiratory epithelial cell state (A) and wild-type immune cell recruitment (B) to study the effect of knockouts such as FADD in respiratory epithelial cell apoptosis (C) or p38 in immune cell recruitment (D).

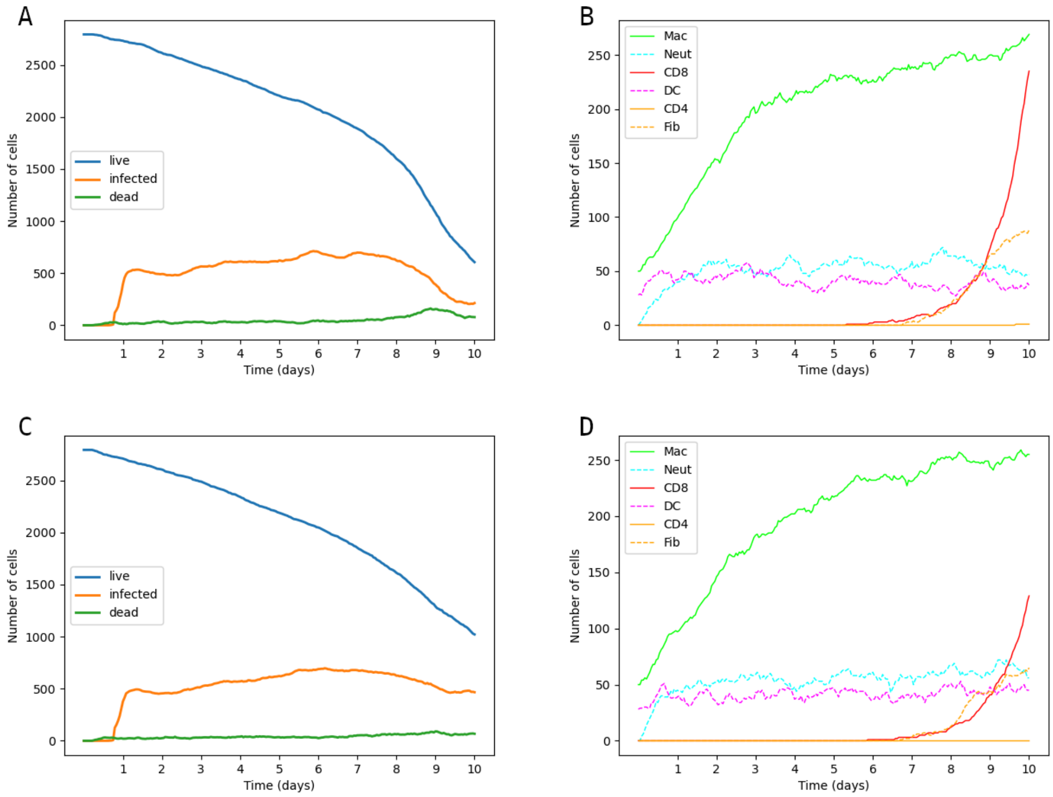

Fig. S14.

Drug targets derived from the multimodal omics data analysis and corresponding disease mechanisms in C19DMap repository.

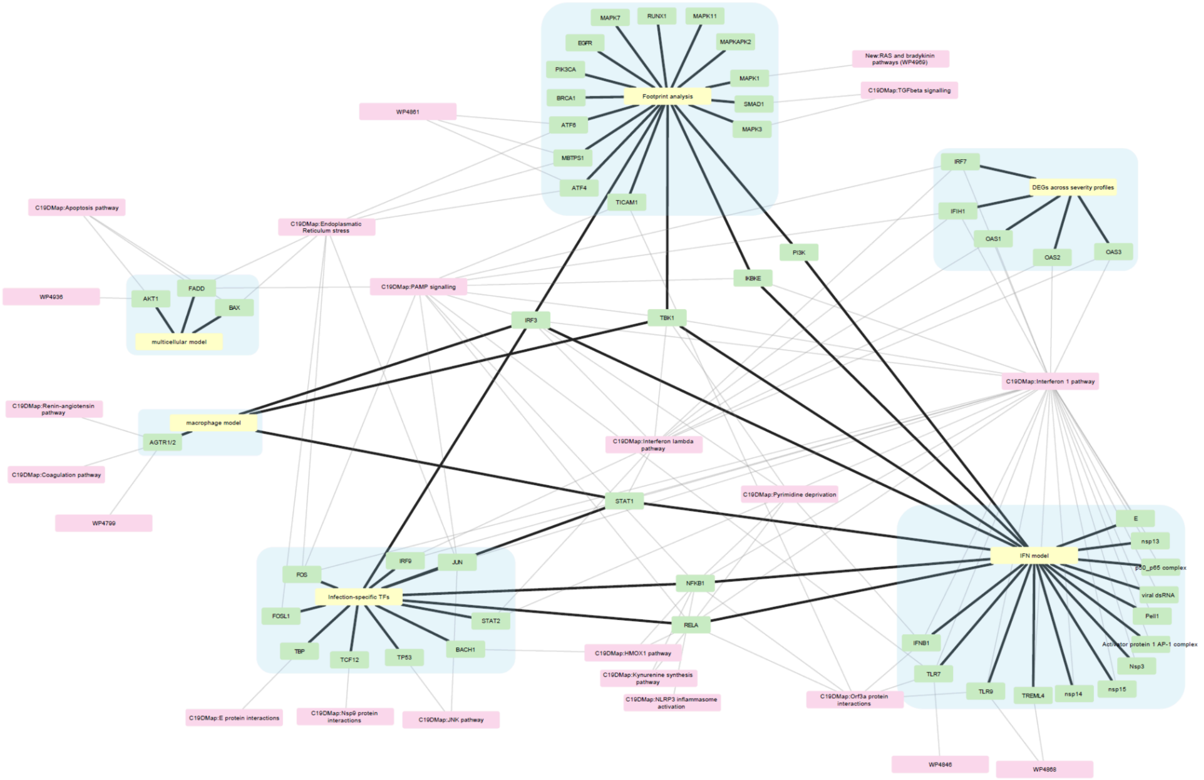

Fig. S15.

CAP score across different populations and stratified by sex. The heatmap displays the CAP score for each gene of the 79 genes computed for each different population and sex. The populations tested are afr: African/African American, eas: East Asian, asj: Ashkenazi Jewish, nfe: non-Finnish European, fin: Finnish European, sas: South Asian, amr: Latino/Admixed American.

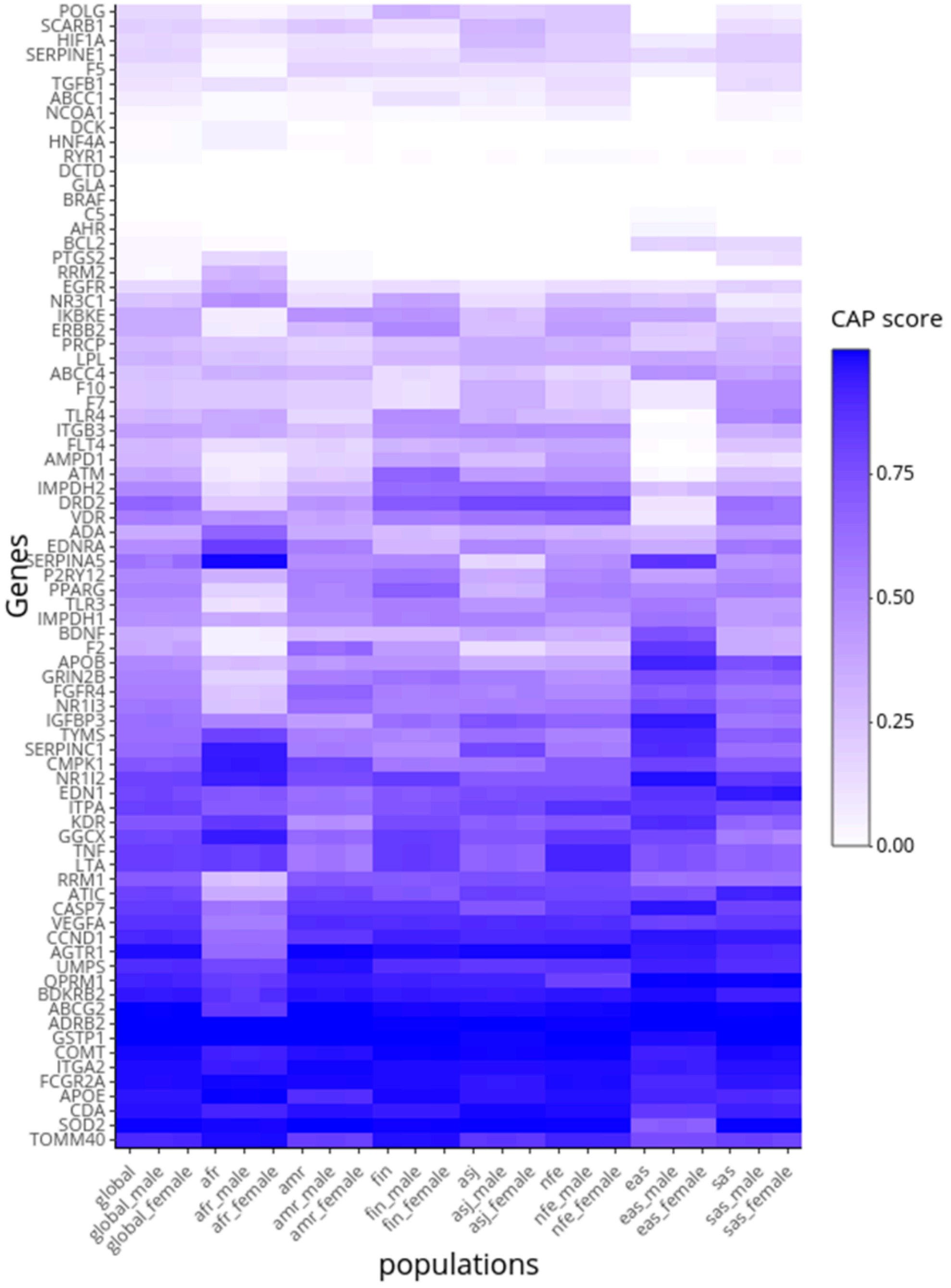

Fig. S16.

DRP score for the set of 52 drugs was computed for each population and sex. The populations tested are afr: African/African American, eas: East Asian, asj: Ashkenazi Jewish, nfe: non-Finnish European, fin: Finnish European, sas: South Asian, amr: Latino/Admixed American.

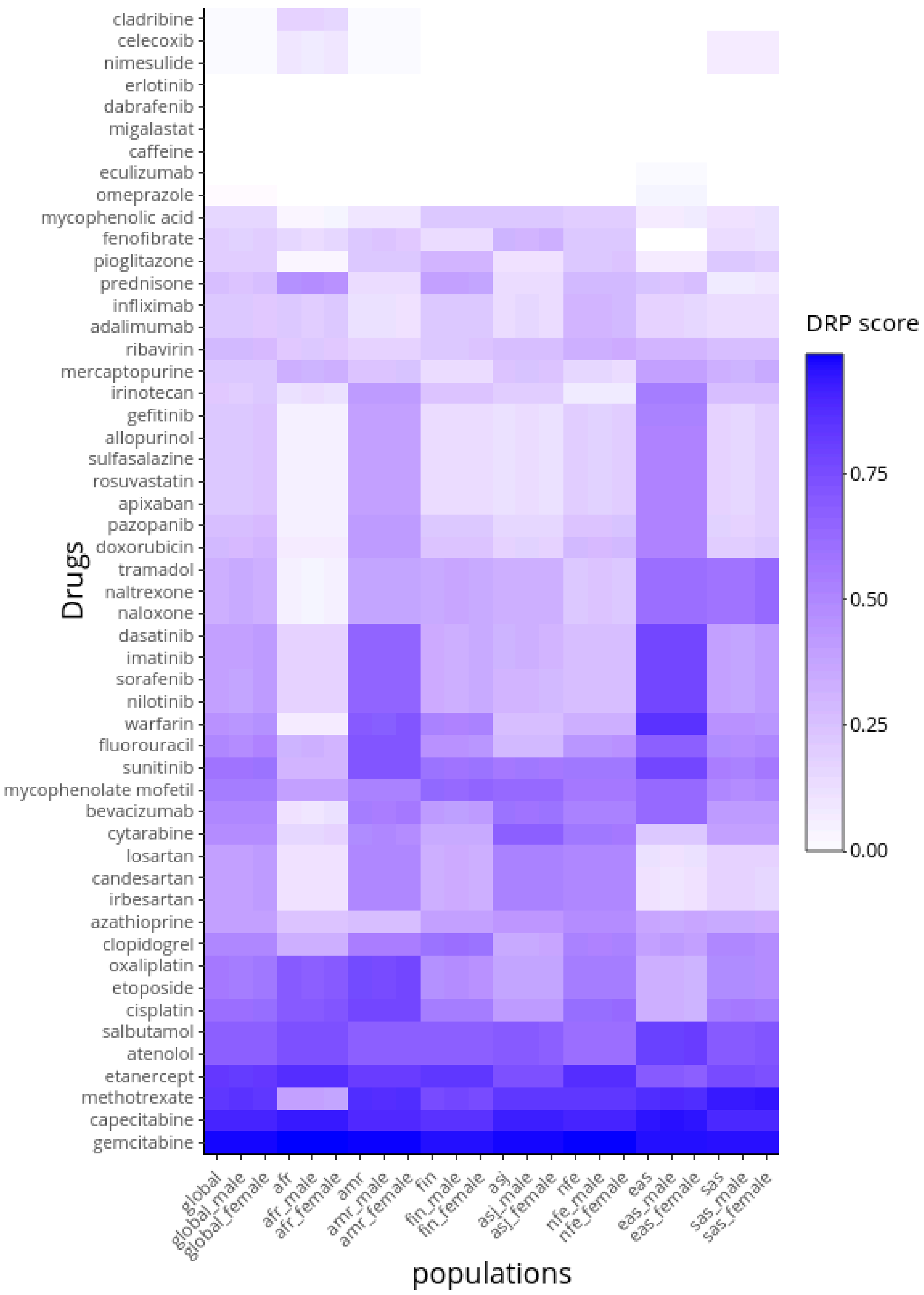

Fig. S17.

C19DM-Neo4j data model: an example of a protein phosphorylation process. For illustration purposes, we show a single process from the Endoplasmic Reticulum Stress map: DDIT3 phosphorylation activated by MAPK14. Nodes represent macromolecules (yellow), processes (blue) and state variables (red). Edges represent relationships: CONSUMES (reactant-process relationship), PRODUCES (process-product), CATALYZES (process-catalyzer), HAS_STATE_VARIABLE (macromolecule-state_variable). Properties of nodes are shown as text near nodes. Entries of the HGNC database and PubMed IDs for supporting references. The C19DM-Neo4j database and the Cypher query language offer flexible querying of the COVID-19 Disease Map resource.

Cypher query example: process phosphorylation

```
MATCH path1 = (product_sv:StateVariable) <- [:HAS_STATE_VARIABLE] -
(product:Macromolecule) <- [:PRODUCES] - (process:Process) -
[:CONSUMES] -> (reactant:Macromolecule) - [:HAS_STATE_VARIABLE] ->
(reactant_sv:StateVariable)
WHERE reactant.label = product.label AND reactant_sv.value IS NULL AND
product_sv.value = "P" AND (product_sv.variable IS NULL AND
reactant_sv.variable IS NULL AND product_sv.order = reactant_sv.order OR
product_sv.variable = reactant_sv.variable)
OPTIONAL MATCH path2 = (catalyzer:Macromolecule) - [:CATALYZES] ->
(process)
OPTIONAL MATCH path3 = (catalyzer) - [:HAS_STATE_VARIABLE] ->
(catalyzer_sv:StateVariable)
RETURN path1, path2, path3
```

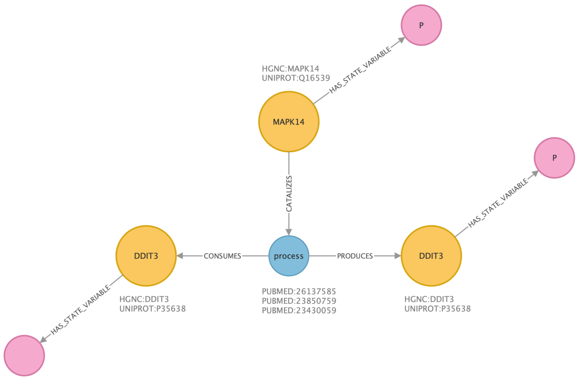

Table S1.

Highlighted C19DMap diagrams, including deregulated kinases and TFs, identified in A549 cells using the footprint analysis.

A549 cells	
**C19DMap diagram**	**hgnc_carnival**
Interferon 1 pathway	IRF3, TBK1, IKBKE
TGFbeta signaling	MAPK3, SMAD1
PAMP signaling	TICAM1, IKBKE, IRF3, TBK1
Pyrimidine deprivation	IRF3, TBK1
Orf3a protein interactions	TICAM1
Endoplasmic Reticulum stress	ATF6, ATF4, MBTPS1
Interferon lambda pathway	IRF3, TBK1

Table S2.

TFs that regulate DEGs in SARS-CoV-2-infected NHBE and A549 cells; "Common" indicates TFs detected in both cell types; TFs in red are those present in the C19DMap.

A549 only			Common	NHBE only	TFs present in C19DMap	C19DMap pathways
E2F4	MYC	E2F1	TP53	STAT2	IRF3	Interferon 1 pathway<br>PAMP signaling<br>Interferon lambda pathway
THAP11	ZNF263	MAZ	STAT1	REL	BACH1	HMOX1 pathway
ZBTB7A	TEAD1	KLF5	FOS	FOSL1	TBP	E protein interaction<br>TGFbeta signaling
NFYB	MEF2A	FOXP1	ESR1	IRF9	TCF12	Nsp9 protein interactions
AR	MXI1	TBP	RELA	PPARA	TP53	JNK pathway
IRF3	KLF9	E2F6	NFKB1	CEBPB	STAT1	Interferon 1 pathway<br>Interferon lambda
SREBF1	MAX	ATF3	KLF6	RBPJ	FOS	Interferon 1 pathway<br>PAMP signaling

SP1	YY1	GABPA	JUN	GATA3	RELA	Interferon 1 pathway<br>PAMP signaling<br>Pyrimidine deprivation<br>Orf3a protein interactions<br>HMOX1 pathway<br>Kynurenine synthesis pathway
SNAPC4	NRF1	NR2C2	-	-	NFKB1	Interferon 1 pathway<br>PAMP signaling<br>Pyrimidine deprivation<br>Orf3a protein interactions<br>HMOX1 pathway<br>Kynurenine synthesis pathway
RUNX2	PRDM14	SIX5	-	-	JUN	Interferon 1 pathway<br>JNK pathway<br>PAMP signaling
MYCN	ZBED1	TEAD4	-	-	STAT2	Interferon 1 pathway<br>Interferon lambda pathway
BACH1	FOXP2	TFDP1	-	-	IRF9	Interferon 1 pathway<br>Interferon lambda pathway
THAP1	CUX1	TCF12	-	-	FOSL1	Endoplasmic reticulum stress
EBF1	ZNF143	MYBL2	-	-		
FOXO3	HBP1	SP2	-	-		
KLF13	BCL6	HIF1A	-	-		
ZEB2	MBD1	ZFX	-	-		
CREB3L1	ARID2	-	-	-	-	-

Table S3.

Differential cell type-specific gene expression between COVID-19 patients and healthy controls. COVID-19 cell sample size (%) represents the absolute count and percentage of lung epithelium cell types in COVID-19 patients-derived samples; Overexpressed DEGs and Downregulated DEGs represent numbers of overexpressed and downregulated DEGs, respectively, in COVID-19 patients vs. healthy controls. 26 COVID-19 overexpressed DEGs were shared between all lung epithelium cell types.

	**COVID-19 cell sample size (%)**	**Overexpressed DEGs**	**Downexpressed DEGs**
**Ciliated cells**	952 (13.5%)	61	93

**Alveolar cells type 1**	139 (5.7%)	183	81
**Basal cells**	469 (10.1%)	166	77
**Secretory cells**	237 (6%)	83	42
**Suprabasal cells**	958 (27.3%)	94	116

Table S4.

26 DEGs shared among all detected lung epithelial cell types. The genes in red belong to Type I interferon induction and signalling pathway in C19DMap (WikPathway WP4868).

ATP6V0C	MX1
OAS1	SRGN
MX2	IFIT2
IFI44L	IFIT3
OAS3	IFI44
EPSTI1	GBP1
IFITM1	CCL2
OAS2	RSAD2
XAF1	IRF7
IFIT1	MT-ATP8
ISG15	NME2
SAMD9	IFI6
TAOK1	STAT1

Table S5.

Significant pathways-circuits activity values after Wilcoxon test comparison between 430 SARS-CoV-2-infected and 54 non-infected individuals. The results were obtained by running CoV-Hipathia web tool with GSE152075 dataset.

Pathway: Effector Circuitname	UP/DOWN	statistic	FDR-p.value	Fold Change	logFC
Nsp9 protein interactions: EIF4H	DOWN	-7.56E+00	6.09E-12	6.29E-01	-6.68E-01
**Interferon lambda pathway: STAT1,STAT2,STAT3**	**UP**	**7.16E+00**	**5.77E-11**	**2.01E+00**	**1.01E+00**

**Interferon 1 pathway: OAS1,OAS2,OAS3**	**UP**	**6.89E+00**	**2.77E-10**	**2.88E+00**	**1.53E+00**
Nsp9 protein interactions: MYCBP2	UP	6.57E+00	1.62E-09	1.26E+00	3.32E-01
Nsp9 protein interactions: COMT	DOWN	-6.54E+00	1.62E-09	4.13E-01	-1.28E+00
Nsp9 protein interactions: DCAF7	UP	5.62E+00	4.65E-07	1.23E+00	2.93E-01
Nsp9 protein interactions: CYB5R3	DOWN	-5.57E+00	5.35E-07	7.47E-01	-4.20E-01
JNK pathway: JUN,JUND	DOWN	-5.47E+00	8.14E-07	5.83E-01	-7.78E-01
Nsp9 protein interactions: MIB1,DLL1	DOWN	-4.83E+00	1.38E-06	1.31E-01	-2.93E+00
E protein interactions: STOML3,ASIC1	DOWN	-5.12E+00	2.10E-06	3.48E-01	-1.52E+00
**Renin-angiotensin pathway: LNPEP**	**UP**	**4.87E+00**	**1.45E-05**	**1.25E+00**	**3.23E-01**
Nsp9 protein interactions: AP2M1	DOWN	-4.56E+00	6.26E-05	8.04E-01	-3.14E-01
E protein interactions: CCNT1,CDK9	DOWN	-4.52E+00	6.73E-05	5.00E-01	-1.00E+00
**Interferon 1 pathway: ISG15**	**UP**	**4.26E+00**	**1.81E-04**	**2.55E+00**	**1.35E+00**
Kynurenine synthesis pathway: AHR	UP	4.28E+00	1.81E-04	1.18E+00	2.35E-01
Nsp9 protein interactions: GFER	DOWN	-4.10E+00	1.98E-04	4.41E-01	-1.18E+00
Nsp9 protein interactions: IMPDH2	DOWN	-4.13E+00	3.10E-04	6.98E-01	-5.19E-01
Renin-angiotensin pathway: MAS1	UP	4.06E+00	3.50E-04	2.45E+00	1.29E+00
Nsp4 and Nsp6 protein interactions: Nsp3:Nsp4:Nsp6	DOWN	-3.73E+00	5.78E-04	5.55E-01	-8.49E-01
E protein interactions: CRB3,PATJ,MPP5	DOWN	-3.69E+00	5.88E-04	4.71E-01	-1.09E+00

Coagulation pathway: MAS1	UP	3.85E+00	5.88E-04	1.28E+01	3.68E+00
HMOX1 pathway: RBX1,KEAP1,CUL3	DOWN	-3.87E+00	6.33E-04	2.73E-01	-1.87E+00
Orf3a protein interactions: HMOX1,ALG5,ARL6IP6	DOWN	-3.46E+00	7.28E-04	2.45E-01	-2.03E+00
Nsp4 and Nsp6 protein interactions: DNAJC11	DOWN	-3.63E+00	8.61E-04	5.67E-01	-8.19E-01
Nsp9 protein interactions: SEPSECS	UP	3.65E+00	1.48E-03	1.66E+00	7.35E-01
Nsp4 and Nsp6 protein interactions: TIMM29,TIMM22,TIMM10B	DOWN	-3.24E+00	1.70E-03	3.85E-01	-1.38E+00
Nsp4 and Nsp6 protein interactions: IDE	DOWN	-3.44E+00	1.77E-03	5.59E-01	-8.40E-01
Nsp9 protein interactions: MRPS5,MRPS2	DOWN	-3.50E+00	2.13E-03	6.66E-01	-5.86E-01
Nsp9 protein interactions: FBLN5,LOXL1	DOWN	-3.22E+00	2.68E-03	3.35E-01	-1.58E+00
Apoptosis pathway: CASP7	UP	3.37E+00	3.66E-03	1.34E+00	4.22E-01
Nsp9 protein interactions: COPS6,EDN1	DOWN	-3.00E+00	4.52E-03	4.26E-01	-1.23E+00
Nsp9 protein interactions: POLR2A,GTF2F2,POLR2G	DOWN	-2.99E+00	9.43E-03	5.54E-01	-8.53E-01
Nsp9 protein interactions: BAG6,EDN1	DOWN	-2.68E+00	1.51E-02	8.07E-01	-3.10E-01
Nsp9 protein interactions: AP2A2	DOWN	-2.74E+00	2.65E-02	8.02E-01	-3.18E-01
Nsp9 protein interactions: RALA	UP	2.70E+00	2.84E-02	1.31E+00	3.87E-01
Nsp9 protein interactions: SBNO1	UP	2.64E+00	3.18E-02	1.06E+00	8.03E-02
Nsp9 protein interactions: DDX10	UP	2.64E+00	3.18E-02	1.28E+00	3.53E-01

Nsp9 protein interactions: UBQLN4,EDN1	DOWN	-2.37E+00	3.18E-02	9.24E-01	-1.13E-01
PAMP signalling: ITCH	UP	2.62E+00	3.26E-02	1.08E+00	1.18E-01
Nsp9 protein interactions: ZNF503	DOWN	-2.47E+00	3.39E-02	6.31E-01	-6.63E-01
Nsp9 protein interactions: CCDC86	DOWN	-2.53E+00	3.42E-02	7.05E-01	-5.05E-01
Nsp9 protein interactions: TCF12	UP	2.53E+00	3.97E-02	1.04E+00	6.07E-02
Nsp9 protein interactions: MAT1A,MAT2A,MAT2B	DOWN	-2.36E+00	4.34E-02	6.69E-01	-5.81E-01
Nsp9 protein interactions: COPS2,COPS4,COPS5	DOWN	-2.44E+00	4.57E-02	6.84E-01	-5.49E-01
Nsp9 protein interactions: MEPCE,LARP7	DOWN	-2.45E+00	4.57E-02	8.06E-01	-3.11E-01
Nsp4 and Nsp6 protein interactions: ATP6AP1	DOWN	-2.41E+00	4.99E-02	8.33E-01	-2.63E-01
Nsp9 protein interactions: LARP4B	UP	2.39E+00	5.07E-02	1.03E+00	4.31E-02
Nsp9 protein interactions: ZNF503,DCAF7	DOWN	-2.27E+00	5.07E-02	7.46E-01	-4.23E-01

Table S6.

Simulation scenarios and model behavior.

No	Biological Scenarios	Simulation results	Model performance (agreement/ partial agreement/ disagreement)
**1**	An unbalanced immune response, characterized by a weak production of type I interferons (IFN-Is) and an exacerbated release of proinflammatory cytokines, contributes to the severe forms of the COVID-19 disease. [PMID:	When all of the viral components are active, the inflammation phenotype is activated.	Agreement

	32726355]		
**2**	SARS-CoV-2-inhibition of type I IFN responses in infected cells leads to delayed or suppressed type I IFN responses, allowing the virus to replicate unchecked and induce tissue damage.<br>[PMID: 33619493, 33097660]<br>SARS-Cov-2 is a poor inducer of IFN-I response *in vitro* and in animal models compared to other respiratory RNA viruses.<br>[PMID: 32726355]<br>Multiple viral structural and non-structural proteins antagonize interferon responses, contributing to inflammation.<br>[PMID: 33619493, 32346093]<br>The suppression of interferon signalling is a mechanism widely used by SARS-CoV-2 in diverse tissues to evade antiviral innate immunity, and targeting the viral mediators of immune evasion may help block virus replication in patients with COVID-19. [PMID: 33140044]	When all of the viral components are active, the type 1 interferon immune response is inactive while the inflammation phenotype gets activated. We observe the inflammation reduction and activation of ISG expression_antiviral response_phenotype after deactivating all of the viral components.	Agreement
**3**	The viral ORF6, ORF8 and nucleocapsid proteins were potent inhibitors of the type I interferon signalling pathway, a key component for the antiviral response of host innate immunity. All three proteins strongly inhibited type I interferon (IFN-β) and NF-κB-responsive promoters. [PMID: 32589897]	In the presence of the ORF6 and ORF8 complex, and nucleocapsid protein (N), NFkB pathways and the type I interferon secretion are inactive.	Agreement
**4**	SARS-CoV-2 desensitizes host cells to interferon by inhibiting the JAK-STAT pathway.<br>[PMID: 33140044]	By activating the viral components, we observe the deactivation of STAT1 and STAT2_phosphorylated, which are part of the JAK/STAT pathway.	Agreement
**5**	The antiviral response is intensified by various signalling factors, including sensors and transcriptional regulators, which are themselves ISGs induced by ISGF3 and/or directly by the IRF3/IRF7 transcriptional activators. [PMID: 32726355]<br>The IRF3 gene is highly expressed in the activated CD4+ T cells of COVID-19 patients.[PMID: 32783921]	Interferon regulatory factor 3 (IRF3 homodimer complex_nucleus) is activated by adding Azithromycin drug to the viral components including orf7a/b, orf3a, viral dsRNA, Nsp1, Nsp13, and E protein.<br>(Initial condition of the IRF3 affects complex formation.)	Agreement

**6**	Several interferon (IFN)-stimulated genes (ISGs; including ISG15, IFI44, IFI44L, and RSAD2) were specifically upregulated in PBMCs from COVID-19 patients, enhancing antiviral and immune-modulatory functions after viral infection. [PMID: 32783921]	When all of the viral components are active, the ISG expression_antiviral response_phenotype is inactivated; however, adding the Azithromycin_drug increases the ISG antiviral response.<br>ISG15 ubiquitin-like modifier is inactive in this condition; however, blocking orf6 and activating STAT1/2_IRF9_complex can upregulate the expression of the ISG15 by affecting ISRE_complex pathways.	Partial agreement
**7**	Inhibition of SCoV2-PLpro with GRL-0617 impairs the virus-induced cytopathogenic effect, maintains the antiviral interferon pathway and reduces viral replication in infected cells. [PMID: 32726803]	When all of the viral components are active, the type 1 interferon response is inactive; By activating the GRL0617 drug, we didn't observe any effect on the antiviral interferon pathways or reduction of viral replication. The model suggests that applying GRL-0617 alone cannot impact this pathway.	Disagreement

Table S7.

A list of 54 drug targets identified from the integrative and computational modeling analyses of omics data and C19DMap diagrams.

MAPK11	STAT1	TREML4
SMAD1	FOS	TBK1
TICAM1	JUN	ARL6IP6
TBK1	RELA	CASP7
IKBKE	NFKB1	LNPEP
IRF3	STAT2	HMOX1
ATF4	IRF9	FADD
ATF6	BACH1	AKT1
MBTPS1	TBP	ALG5
TP53	TCF12	AGTR1/2
STAT3	IFIH1	EGFR
ISG5	OAS1	KEAP1
JUND	OAS2	CUL3
AHR	OAS3	E
FOSL1	IRF7	nsp15
MAS1	BAX	nsp14
RBX1	IFNB1	nsp3
TLR9	TLR7	nsp13

Tables S8 and S9 provided as a separate excel file

Table S10.

Top ranking instances across all pathways of the C19DMap bipartite graph.

Ranking	Aggregated centrality value	Map species
1	3.088928834	UNIPROT:P0DTD1<br>**Replicase polyprotein 1ab SARS COV2**
2	3.060313914	UNIPROT:P0DTC9<br>**Nucleoprotein NCAP_SARS2**
3	3.045092464	UNIPROT:Q9BYF1<br>**Angiotensin-converting enzyme 2**
4	2.974406697	UNIPROT:P0DTC3<br>**ORF3a protein SARS COV2**
5	2.960697776	UNIPROT:P59632

		**ORF3a protein SARS COV**
6	2.926930595	UNIPROT:P59637<br>**Envelope small membrane protein SARS COV**
7	2.921492419	UNIPROT:P59595<br>**Nucleoprotein SARS COV**
8	2.91891734	**Nsp6**
9	2.917060121	UNIPROT:P0DTC4<br>**Envelope small membrane protein SARS COV2**
10	2.894981602	UNIPROT:P0DTC1<br>**Replicase polyprotein 1a SARS COV2**

Table S11.

Top ranking instances by the aggregated centrality values within the Interferon type I pathway.

Aggregated centrality value	Map species
2.99852173	UNIPROT:Q14653<br>**IRF3**
2.90197689	**Nsp3**
2.833324697	UNIPROT:Q04206;UNIPROT:P19838<br>**RELA:NFKB1**
2.770348427	**Nsp15**
2.673571447	UNIPROT:Q7Z434<br>**MAVS**
2.568232753	**Viral_dsRNA**
2.522809862	UNIPROT:P01574<br>**IFNB**
2.425625918	**Orf6**
2.393485439	UNIPROT:Q14164;UNIPROT:Q9UHD2;UNIPROT:Q13114;UNIPROT:Q92844<br>**IKBKE:TBK1:TRAF3:TANK**
2.341295307	UNIPROT:O15455<br>**TLR3**

Table S12.
Top ranking instances by the aggregated centrality values within the Interferon lambda pathway.

Aggregated centrality value	Map species
2.647094921	UNIPROT:Q14653<br>**IRF3**
2.614378989	**IFN_III**
2.324754461	UNIPROT:P10914<br>**IRF1**
2.313925511	UNIPROT:Q8IZI9;UNIPROT:Q8IZJ0;UNIPROT:Q8IU54;UNIPROT:K9M1U5<br>**IFNL3: IFNL2:IFNL1:IFNL4**
2.122800448	UNIPROT:Q8IU57;UNIPROT:P23458;UNIPROT:P29597<br>**IFNLR1:JAK1:TYK2**
2.027010129	**dsRNA**
1.923644106	UNIPROT:O95786<br>**RIG-I**
1.814816851	UNIPROT:O14920;UNIPROT:O15111;UNIPROT:O95786;UNIPROT:Q9UHD2;UNIPROT:Q7Z434<br>**IKK:TBK1:RIG-I:MAVS**
1.790330986	UNIPROT:O95786;UNIPROT:Q7Z434<br>**RIG-I:MAVS**
1.537042334	UNIPROT:Q7Z434;UNIPROT:O95786<br>**MAVS:RIG-I**

Table S13.
Top ranking instances by the aggregated centrality values within the Apoptosis pathway.

Aggregated centrality value	Map species
2.790670304	UNIPROT:Q14790<br>**CASP8**
2.575114314	UNIPROT:P55211<br>**CASP9**

2.46327704	**Apoptosis**
2.33125067	UNIPROT:P59637<br>**Envelope small membrane protein SARS**
2.208875087	UNIPROT:P42574<br>**CASP3**
2.208875087	UNIPROT:P55210<br>**CASP7**
2.179943105	UNIPROT:Q13158<br>**FADD**
2.146130422	UNIPROT:P31749<br>**AKT1**
1.796281512	UNIPROT:P99999<br>**CYCS**
1.775255449	UNIPROT:P55957<br>**BID**

Table S14.

Top ranking instances by the aggregated centrality values within the Coagulation pathway.

**Aggregated centrality value**	**Map species**
3.024512997	**SARS_CoV_2_infection**
2.768981505	UNIPROT:P00734<br>**Prothrombin/ Thrombin (F2)**
2.742875284	UNIPROT:P00747<br>**Plasminogen (PLG)**
2.664962377	UNIPROT:P13671;UNIPROT:P07357;UNIPROT:P01031;UNIPROT:P10643;UNIPROT:P07360;UNIPROT:P02748;UNIPROT:P07358<br>**C6:C8A:C5:C7:C8G:C9:C8B (C5b-9)**
2.65809911	UNIPROT:P30556<br>**AGTR1**
2.657606153	UNIPROT:P0C0L5<br>**C4B**
2.631358595	**Thrombosis**

2.549145423	UNIPROT:P00750<br>**PLAT**
2.540683322	UNIPROT:P04070<br>**PROC**
2.432797266	UNIPROT:P05121<br>**SERPINE1**

Table S15.

Top ranking instances by the aggregated centrality values within the Renin-Angiotensin pathway.

**Aggregated centrality value**	**Map species**
2.952636737	UNIPROT:P30556<br>**AGTR1**
2.876342802	UNIPROT:Q9BYF1<br>**ACE2**
2.760902828	UNIPROT:P12821<br>**ACE**
2.56692845	UNIPROT:P04201<br>**MAS**
2.382326458	UNIPROT:P50052<br>**AGTR2**
2.296031373	UNIPROT:P0DTC2;UNIPROT:P59594<br>**SPIKE_SARS2: SPIKE_SARS**
2.237870739	**angiotensin_A**
2.217315056	**angiotensin_1-12**
2.141851032	**angiotensin_3-7**
2.135741406	UNIPROT:P08473<br>**EPN**

Table S16.

11 out of the 54 identified targets that rank in the top 30% instances of the aggregated network by aggregated centrality values.

Protein / Gene	*Node label*	*Rank*	*Aggregated centrality value*
HMOX1	*UNIPROT09601*	*32*	*2,74319632*
STAT2	*UNIPROT52630*	*92*	*2,4594734*
RBX1	*UNIPROT62877*	*98*	*2,43757737*
ATF6	*UNIPROT18850*	*129*	*2,33493298*
TREML4	*UNIPROT:Q6UXN2*	*145*	*2,28702511*
ATF4	*UNIPROT18848*	*385*	*1,90123974*
TCF12	*UNIPROT:Q99081*	*512*	*1,84628427*
OAS3	*UNIPROT:Q9Y6K5*	*548*	*1,80576535*
OAS2	*UNIPROT29728*	*549*	*1,80576535*
OAS1	*UNIPROT00973*	*550*	*1,80550953*
ISG15	*UNIPROT05161*	*561*	*1,78758416*

Table S17.

Number of drug candidates targeting transcription factors detected; "Common Transcription Factor" represents transcription factors detected for both NHBE and A549 cell types listed in Table 1; "External Clinical Trials" represents drugs already in external clinical trials for COVID19 designated in DrugBank.

	External Clinical Trials	Not External Clinical Trials
Common Transcription Factor	47	160
Not Common Transcription Factor	36	103
